# The Perpetual Cycle of Racial Bias in Healthcare and Healthcare Education: A Systematic Review

**DOI:** 10.1007/s40615-025-02417-6

**Published:** 2025-04-16

**Authors:** Vittoria Sorice, Gerri Mortimore, Mark Faghy, Rossella Sorice, Davinder Tegally

**Affiliations:** 1 College of Health, Psychology and Social Care, University of Derby, Derby, UK; 2 Emergency and Acute Medicine, Chesterfield Royal Hospital NHS Foundation Trust, Chesterfield, UK; 3 College of Science and Engineering, University of Derby, Derby, UK; 4 University of Naples Federico II, Naples, Italy; 5 Medicines and Healthcare Products Regulatory Agency, London, UK; 6 Care Quality Commission, Sheffield, UK

**Keywords:** Racial bias, Healthcare disparities, Health equity, Healthcare education, Cultural competence

## Abstract

**Objectives:**

This systematic review aimed to identify and categorise racial bias in healthcare and education; evaluate their impact on healthcare practitioners, students, and patients; and explore strategies to reduce these biases and improve health equity.

**Method:**

A systematic review was conducted following PRISMA guidelines to investigate racial bias in healthcare and healthcare education. Databases searched included PubMed, Cochrane, EMBASE, and CINAHL. Identification of additional papers was completed by employing backward and forward snowballing techniques. Rigorous interprofessional multi-reviewer screening and data extraction processes were performed. Thematic analysis was conducted inductively and collaboratively refined, with disagreements resolved through discussion and a third reviewer confirming resolutions as an additional quality assurance measure.

**Results:**

From an initial pool of 1634 records, 45 studies were included in the final review. The studies employed various designs, primarily cross-sectional, with most conducted in the United States of America. Five themes emerged: disparities in healthcare access and/or provision, perceived discrimination and/or medical mistrust, provider bias and/or stereotyping, disparities in education and training, and healthcare literature disparities.

**Conclusions:**

The findings suggest significant racial disparities across multiple medical specialties, including maternal and infant healthcare, chronic disease management, and emergency care. The review also highlights the underrepresentation of racial minorities in medical imagery and educational materials, contributing to implicit bias and inadequate training for healthcare providers. Overall, the five identified themes appear interconnected, forming a self-reinforcing cycle of racial bias in healthcare and education.

**Supplementary Information:**

The online version contains supplementary material available at 10.1007/s40615-025-02417-6.

## Introduction

Racial bias in healthcare and healthcare education is a pervasive and deeply ingrained issue that significantly affects the quality, accessibility, and equity of care provided to racial and ethnic minorities. These groups often experience systemic barriers to accessing care and receive lower-quality healthcare services compared to their White counterpart [1, 2]. These biases are deeply embedded in systemic inequalities across healthcare institutions [3]. They manifest as disparities in patient treatment, limited access to critical cures, poorer health outcomes for minorities compared to White populations, and inadequate quality monitoring strategies to detect and address these issues [4, 5]. Understanding these dynamics is critical for addressing the barriers faced by marginalised populations in healthcare systems worldwide.

Globally, racial disparities in healthcare outcomes are striking. In the United States of America (USA), Black women are three times more likely to die from pregnancy-related complications than White women [6], while racial minority infants face an infant mortality rate more than twice that of White infants [7]. Similarly, in the United Kingdom (UK), Black maternal mortality rates are almost four times higher than those of White women, and Black children experience a child death rate more than double that of White British children [8, 9]. These patterns extend beyond maternal and infant health outcomes to encompass various medical specialties. Minority groups often face significant barriers in accessing healthcare, influenced by systemic racism and social determinants, and these challenges were exacerbated during the SARS-CoV- 2 pandemic, impacting timely diagnosis, treatment, and immunisation [10, 11]. The broader issue of racial disparities in healthcare is reflected in research consistently demonstrating that Black patients receive lower-quality care compared to their White counterparts. This includes less frequent use of supportive medications in palliative care, with Black patients having an adjusted odds ratio (aOR) of 0.84 for opioid utilisation compared to non-Hispanic White patients with pancreatic cancer [12]. Black patients are also less likely to undergo significant surgical interventions, such as preoperative MRI for breast cancer, with a lower utilisation rate of 29.85% compared to 33.10% for White patients (*p* < 0.001) [13]. Moreover, Black patients experience poorer pain management across both cancer and non-cancer care settings [14]. In cancer related care, only 35% of minority patients receive adequate analgesia, compared to 50% of their non-minority counterparts [15]. Similarly, in fracture-related cases, 57% of minority patients received appropriate analgesia in emergency settings, compared to 74% of non-minority patients [16].

In terms of psychiatric support, Black patients also have lower use of antidepressants (aOR, 0.56) and anxiolytics (aOR, 0.78) compared to non-Hispanic White patients [12]. In surgical interventions, Black patients are more likely to undergo open surgeries rather than less invasive procedures, such as transcatheter interventions for congenial heart diseases, with an aOR of 1.49 for open surgeries [17]. In terms of hospitalisation outcomes, Black patients with metastatic breast cancer have higher in-hospital mortality rates (aOR 1.25) [18]. These disparities highlight systemic inequalities in healthcare access and quality that need to be addressed to ensure equitable care for all patients.

The SARS-CoV- 2 pandemic further exacerbated these disparities. Minority groups experienced disproportionately higher rates of infection, severe illness, and mortality due to systemic barriers in accessing timely diagnosis and treatment [19, 20]. These challenges underscored how implicit bias intersects with social determinants of health to amplify inequities during global health crises.

Implicit bias plays a central role in perpetuating these disparities. Unlike explicit prejudices, implicit biases operate unconsciously, influencing perceptions and behaviours without individuals’ awareness [21]. They are embedded in both individual interactions and institutional practices, often manifesting as microaggressions or subtle forms of discrimination [22]. Research has consistently demonstrated that implicit bias among healthcare providers contributes to disparities in care. For instance, in the same realm of pain management, studies have shown that false beliefs about biological differences between black and white individuals persist among medical students and residents [14]. Specifically, a substantial number of medical students and residents endorsed misconceptions such as the belief that Black people’s skin is thicker than White people’s, with 25% of residents to 40–42% of first- and second-year medical students holding this false belief, which can influence clinical decision-making regarding treatments [14].

Racial bias also permeates healthcare education. Curricula frequently lack comprehensive discussions on cultural competency or the role of socioeconomic determinants of health, perpetuating biases [23, 24]. Educational resources often underrepresent minority populations; for instance, Louie and Wilkes [25] examined racial and skin tone representation in medical textbooks, revealing a disparity where light skin tones were overrepresented (74.5%) and dark skin tones underrepresented (4.5%), despite proportional racial diversity for the USA. This lack of representation reinforces stereotypes and limits providers’ ability to diagnose conditions across diverse populations. Furthermore, minority students in healthcare sectors typically face challenges that limit their educational opportunities and professional paths [26, 27]. These challenges contribute to a lack of diversity among healthcare professionals and perpetuate inequities in patient care.

While systematic reviews have explored racial bias in healthcare [28–31], they largely focused on specific health outcomes or regions, often overlooking the broader educational and organisational factors that contribute to healthcare inequities. Numerous studies have highlighted the pervasive issue of racial bias within healthcare systems. Hall et al. [28] reviewed the impact of implicit racial bias among healthcare professionals, revealing that such biases affect patient-provider interactions and health outcomes, particularly for people of ethnic minorities. Similarly, FitzGerald and Hurst [29] documented implicit bias among healthcare providers, emphasising the need for further research and interventions to address these biases. Paradies et al. [30] conducted a meta-analysis, finding racism linked to negative mental health and poorer general and physical health outcomes. Additionally, Ricks et al. [31] highlighted the lack of effective anti-racist training for healthcare workers post-licensure, calling for a comprehensive shift toward anti-racism in healthcare education, practice, and research. These results collectively emphasise a need for systematic investigations that integrate data across multiple healthcare settings and educational contexts to comprehensively understand the racial bias impact on the healthcare system [3, 32].

This systematic review analyses racial bias in healthcare settings, focusing on its impact on patient care and healthcare providers’ (HPs) education. It aims to identify and categorise racial inequities and biases in healthcare delivery and education, providing a comprehensive summary of how these biases manifest across clinical and educational environments. The review also aims to evaluate how these biases affect diverse stakeholders, including patients, healthcare practitioners, and students, with a focus on uncovering disparities in access to care, providers’ behaviour, and educational outcomes. The findings are organised around five interconnect themes: (1) disparities in healthcare access and/or provision, (2) perceived discrimination and/or medical mistrust, (3) provider bias and/or stereotyping, (4) disparities in healthcare education and/or training, and (5) disparities in healthcare literature. These themes collectively form a self-reinforcing cycle of racial bias in healthcare and education, as illustrated by Supplementary Fig. 1, which serves as the conceptual framework for this review. This cycle often commences with educational disparities in healthcare education, which can lead to the underrepresentation of minorities and inadequate cultural competence among healthcare professionals. This has the potential to perpetuate provider bias, exacerbating healthcare disparities and fostering medical mistrust among minority communities. In turn, this mistrust tends to discourage participation in clinical research, reinforcing educational disparities and perpetuating the cycle of racial bias in healthcare and education.

By examining these overlapping domains and gaps in the literature, this review seeks to inform strategies for breaking the cycle of racial bias in healthcare and education. This will contribute to building a more equitable healthcare system, where healthcare education fosters cultural competence and humility for effective care of diverse populations.

## Methods

### Search Strategy and Selection Criteria

This systematic review synthesised empirical studies evaluating the impact of racial bias in healthcare and education. The review followed the Cochrane Handbook for Systematic Reviews of Interventions [33] and adhered to the Preferred Reporting Items for Systematic Reviews and Meta-Analyses (PRISMA) reporting criteria [34]. The protocol was prospectively registered on PROSPERO (CRD42024518475).

A comprehensive search strategy, guided by the PICo Framework [35], used broad terms like implicit bias, race, ethnicity, healthcare and education (Supplementary Table 1). The search covered databases including PubMed, COCHRANE, EMBASE, CINAHL, and King’s Fund Library. This was executed up to the 5 th of March 2024, with a subsequent repetition before data analysis, which produced no additional findings. Journals focusing on race, justice, social problems, and education were hand-searched and snowballing techniques were applied. A healthcare librarian ensured accuracy.

Articles published in the last 10 years (2014–2024) were included, excluding non-English studies and those with fewer than 100 participants. All primary research designs related to racial bias interventions were included; studies not focused on racism in healthcare or education were excluded. Additionally, the review excluded studies outside healthcare but included those on healthcare-related learning materials to capture educational content potentially promoting racial bias.

An interprofessional multi-reviewer screening process was employed to assess the eligibility of papers based on the predetermined inclusion and exclusion criteria, which included reviewing the titles and abstracts. Two reviewers (RS and VS) assessed each record independently to help reduce individual bias and errors in the study selection process. A third reviewer (DT) was then employed to examine the included records using the same techniques and inclusion criteria to add an additional layer of scrutiny due to the nature of the topic. Any discrepancies were settled through discussion or by consulting a fourth reviewer (GM); however, this step was not necessary.

### Data Analysis

A standardised data extraction form, based on Cochrane guidance [36], was developed to capture bibliographic details, study characteristics, method for assessing racial bias, and relevant findings (Supplementary Table 2). The form was pilot tested for clarity and consistency across the team. Data extraction was performed independently by two reviewers (VS and DT) to minimise errors and bias, with discrepancies resolved through discussion. For each paper, overarching themes were independently identified to capture the key findings and conclusions. This was accomplished through inductive thematic analysis, with two independent researchers (VS and DT) noting themes throughout their review. These initial themes were then discussed, grouped into potential overarching themes, and iteratively refined through collaborative discussion. In total, only four disagreements were found. These were resolved through exhaustive discussion between the two reviewers, who critically examined the data and reached a consensus. A third reviewer (RS) confirmed these resolutions as an additional quality assurance measure. Relevant Critical Appraisal Skills Programme checklist [37] or Mixed-Method Appraisal Tool [38] was then completed for each study by two reviewers (VS and RS).

## Results

The initial search yielded 1593 results and 33 from other sources. Automated tools filtered articles by language and publication date, excluding 570 items, which included duplicates, and 355 non-primary research papers, leaving 701 records for screening. Initial screening eliminated 508 records. Full-text articles were evaluated for eligibility based on their availability, resulting in 133 full-text records that could not be retrieved. Thirty-seven publications satisfied the eligibility criteria, and an additional 8 articles were included employing snowballing techniques, resulting in a total of 45 included studies. The PRISMA flow diagram (Supplementary Fig. 2) denotes the entire research selection process.

The characteristics of the studies included in this analysis, along with the main themes identified, are summarised in Supplementary Table 3 Supplementary Fig. 3 illustrates the various study designs employed and their geographical distribution, while Supplementary Fig. 4 showcases the different population categories represented in each study. Additionally, Supplementary Fig. 5 provides a comprehensive breakdown of the diverse ethnic and racial groups included in the populations of these studies.

The examination of racial and ethnic disparities in healthcare revealed significant inequities affecting minority populations across various medical specialties, with 77.78% of the studies examined reporting data from the USA.

The systematic review identified significant racial and ethnic disparities in healthcare and healthcare education, uncovering five themes: disparities in healthcare access and/or provision, perceived discrimination and/or medical mistrust, provider bias and/or stereotyping, disparities in healthcare education and/or training, and disparities in healthcare literature. Supplementary Fig. 6 illustrates the distribution of studies across these themes.

### Theme 1: Disparities in Healthcare Access and/or Provision

Nineteen studies highlighted persistent racial disparities in healthcare access and/or provision [39–57]. Racial disparities in women’s reproductive health manifested across contraceptive access, perinatal care, and infant mortality [42, 44, 45, 50]. Minority women faced different patterns of contraceptive access and use depending on insurance status [50]. In perinatal care, Black infants were subject to higher mortality rates, though vaginal deliveries offered some protection across all racial groups [44]. The COVID- 19 pandemic further diminished patient autonomy, leading to lower quality of perinatal care for minorities as cases increased [42]. Analysis of extensive birth data revealed that Black infants experience more than double the mortality rate from medical errors compared to White infants, with this disparity increasing over time [45].

In cardiovascular care, significant racial and ethnic disparities persisted across various aspects of treatments and outcomes [41, 43, 48, 55]. African American (AA) children experienced higher waiting list mortality and post-transplant rejection for heart transplant compared to Caucasian children [41]. Non-Hispanic Black and Hispanic adults showed poorer cardiometabolic profiles at all income and insurance levels [48]. Black patients and women were less likely to receive left ventricular assist devices, despite Black patients with lower propensity scores showing better survival outcomes when receiving the devices [43]. In England, Black and South Asian patients had a reduced likelihood of undergoing aortic valve replacement surgery compared to White patients [55]. In hypertension treatment, while providers often adhered to race-based guidelines, Black patients experienced suboptimal hypertension control despite receiving recommended treatments [46, 57].

In cancer care, AA and Native American patients were less likely to receive adequate oncologic resection for colon cancer compared to White patients, attributed to unconscious bias, social inequalities, and limited access to quality healthcare, particularly in rural areas [53].

In emergency care, Sangal et al. [56] found Black and Hispanic patients were often bypassed in the queue, while Longcoy et al. [51] noted higher hospital admission rates for White patients during COVID- 19 hospitalisations. Ali et al. [40] reported that Black patients waited longer for hip fracture care, highlighting racial bias in treatment timing. Additionally, Black and Asian patient experienced higher 7-day readmissions rates among adults discharged form a hospital medicine service [54].

In chronic disease management, studies identified barriers for Non-Hispanic Black and Hispanic diabetic individuals, including delays in care and lower technology use [39, 47]. In pain management, differences in prescription patterns based on race and other demographics were also observed [49, 52].

### Theme 2: Perceived Discrimination and/or Medical Mistrust

Five studies assessed the impact of perceived discrimination and/or medical mistrust [58–62]. Cox et al. [58] reported how sexual minority men from minority backgrounds experience higher levels of medical mistrust compared to their White counterparts, highlighting the intersectionality of race and sexual minority status in healthcare experiences. This medical mistrust extended to clinical research, with perceived discrimination in medical settings inversely related to the likelihood of participating in clinical trials [59].

The impact of discrimination is particularly pronounced in specific healthcare contexts. For instance, patients undergoing methadone maintenance therapy for opioid use disorder reported higher levels of racial discrimination in healthcare settings, with American Indian/Alaska Native participants being disproportionately affected [60]. Similarly, research focusing on American Indian adults with type 2 diabetes revealed that over one-third experienced racial microaggressions in interactions with HPs, correlating with poorer health outcomes such as heart attacks, depressive symptoms, and increased hospitalisation rates [61].

In the context of pain management, veterans from various minority backgrounds reported different aspects of dissatisfaction associated with perceived discrimination, including poor staff interactions and unresolved pain [62].

### Theme 3: Provider Bias and/or Stereotyping

The prevalence of provider bias and stereotyping behaviour among HPs was identified in 14 studies [63–76]. For instance, one study found that implicit and explicit anti-Black and anti-Arab-Muslim prejudices are more pronounced among HPs than the general population [63]. When controlling for demographic factors, these bias became non-significant for physicians while persisting for non-physician HPs [63].

In the Canadian context, a concerning level of explicit bias against Indigenous people was observed among physicians, with 8.3% reporting unfavourable feelings and 25% expressing a preference for White individuals [64]. Significant discomfort among physicians in discussing race and bias was also reported, often evidenced by comments about reverse racism targeting White people [64]. Similarly, in Australia, 60% employees at a cancer centre displayed a preference for White Australians over First Nations Australians [65].

Examination of patient safety reporting systems revealed that female physicians and those from minority backgrounds face disproportionate reporting for minor issues compared to their male and White counterparts [66]. Interestingly, the impact of provider bias on patient outcomes proved complex; one study found no significant correlation between implicit bias and hypertension treatment outcomes among Black and Latino patients [67].

In genetic counselling, both certified professionals and trainees displayed implicit preferences for White over Black patient, despite reporting no explicit prejudice [68]. This discrepancy underlines the potential influence of social desirability on self-reported data [68]. Additionally, the group demonstrated to stereotype Black individuals as more mistrustful [68]. In Belgium’s primary care setting, 82.6% trainee general practitioners exhibited implicit preferences for their own ethnic group while expressing conflicting attitudes toward migrant care needs [69]. Indeed, over 50% of participants believed that migrants should be responsible for adopting host country values [69].

In medical education, a study on over 3500 students training across 49 USA medical schools found that incorporating the Implicit Association Test in the curricula was associated with a decreased in implicit racial bias [70]. Conversely, exposure to negative comments about AA patients increased implicit bias among medical students, while positive interactions decreased it [70]. Further research on assumptions among medical students regarding patients seeking HIV prevention revealed intricate biases influenced by race and gender identity, complicating perceptions of adherence based on patient characteristics [71]. Meanwhile, a randomised controlled study in a Dutch context found that individual factors rather than external messages significantly shaped stereotypes among students and teachers [72].

In emergency medicine, high levels of implicit bias favouring Non-Hispanic Whites among emergency HPs caring for American Indian children were found, with 84% of participants showing some implicit preference for Non-Hispanic Whites, though little difference was seen in clinical vignette responses based on the race of the child described [73]. Hymel et al. [74] extended these insights by examining racial and ethnic disparities in the evaluation and reporting of abusive head trauma (AHT) in children across 18 sites. They found that minority patients were more frequently evaluated and reported for suspected AHT, particularly in lower-risk cases [74]. These disparities were most pronounced at two specific sites, suggesting local practices and implicit bias may significantly influence clinical decision-making [74].

Examination of clinical documentation has also revealed concerning patters in language use and information recording, discovering that Hispanic and Black patients were more likely to be subjected to judgmental language [75]. When this was used, a 21-min reduction in the duration of a home visit was also observed [75]. Similarly, significant disparities were found in the documentation of essential health information (the 4Ms: What Matters, Mobility, Medication, and Mentation) across racial groups in telehealth settings for older adults, with notably lower documentation rates for Asian, Black, and Hispanic patients compared to their White counterparts [76].

### Theme 4: Disparities in Healthcare Education and/or Training

Three studies on differential attainment and awarding gaps in the UK medical education revealed disparities in healthcare education and/or training affecting minority ethnic groups [77–79]. A study involving 3714 students at a single medical programme found a consistent attainment gap between White and Non-White students, with White students more likely to achieve merit or distinction [77]. Notably, this gap widened for Black students as they progressed through their training [77]. Another analysis of over 16,000 students across 33 UK medical schools provided a national perspective, showing significant variations in the awarding gap between different ethnic minority subgroups and across institutions [78]. After controlling for various factors, students from all minority ethnic backgrounds had lower UK Foundation Programme scores compared to White students, with Pakistani students experiencing the largest gap [78].

An examination of Public Health specialty recruitment highlighted how standardised assessments can perpetuate racial disparities in postgraduate training [79]. Candidates from Black and Asian ethnic background and non-UK medical graduates performed worse across all assessment compared to White British applicants, even after adjusting for other factors such as age, sex, and professional backgrounds [79].

### Theme 5: Disparities in Healthcare Literature

Four studies accentuated the underrepresentation of racial and ethnic minorities in medical literature and imagery, raising concerns about the impact on patient care and healthcare education [80–83]. An analysis of over 4100 articles in plastic surgery journals uncovered that only 22% depicted Non-White individuals, despite Non-White people comprising 60 to 70% of the global population and 30% of the USA cosmetic surgery patients [80].

In emergency medicine journals, only 13.6% of images of cutaneous disorders featured dark-skinned individuals, raising concerns about clinicians’ ability to recognise diverse skin conditions [81]. Similarly, Kalantari et al. [82] reported that only 13.6% of images in emergency medicine textbooks comprised Non-White individuals, with only 11.2% depicting females.

Expanding the scope, Rana et al. [83] focused on lupus imagery across rheumatology, dermatology, and internal medicine resources, revealing that 56.4% of images depicted light skin tones, despite lupus disproportionately affecting racial minorities.

## Discussion

This systematic review examined the complex aspects of racial bias in healthcare and education, highlighting a cycle that perpetuates disparities. This conceptual model, derived from observing overlapping themes across numerous studies, emphasises how various forms of racial bias perpetuate and reinforce each other, creating a self-sustaining cycle that adversely affects healthcare delivery and education (Supplementary Fig. 1).

The cycle begins with disparities in healthcare education and training. These educational inequities manifest in various ways, from underrepresentation of minorities in healthcare education to biased curricula and lack of cultural competence training [64, 77–79]. The scarcity of diverse learning materials [80–84] and barriers to accessing specialised training [79, 85, 86] result in inadequate preparation for HPs [3]. Healthcare students (HSs) may also be exposed to bias [68, 70], which can hinder their learning [87], career advancement, or even lead to higher attrition rates [88]. These disparities limit opportunities for HSs, perpetuating attainment gaps and hindering the diversification of the healthcare workforce itself.

Inadequate education and exposure to biased peers, educators, and HPs result in a workforce that is ill-prepared to address diverse populations’, perpetuating provider bias and stereotyping. Racial bias among HPs is well-documented across different countries and healthcare contexts [63–76] with higher levels of implicit and explicit prejudice among HPs compared to the general population [63]. The perpetuation of provider bias is rooted in several interconnected factors, including the historical context of colonialism and systemic racism [65, 89] and the persistence of a Eurocentric or White-centric curriculum [64, 70, 84]. This is exacerbated by insufficient educational resources [80–83], inadequate cultural awareness training [64, 65], and lack of peer diversity [66, 70]. Underrepresentation in healthcare leadership and academia perpetuates these biased perspectives and practices [64–66, 70].

Provider bias in healthcare manifests in various ways, from clinical decision-making to communication, creating barriers to equitable care for marginalised groups [66, 67, 73–76]. They manifest across various medical specialties and patient populations, significantly impacting health outcomes for minority groups [39–57]. It affects professional relationships as microaggressions and implicit bias create a hostile work environment, particularly for minority staff, leading to increased stress, burnout, and job dissatisfaction among HPs [90], often resulting in higher turnover rates [88, 91]. This demonstrates that while increasing diversity is a positive step, it cannot fully address these issues without broader systemic change.

Patients who consistently experience treatment bias and care disparities are more likely to perceive discrimination within the healthcare system leading to increased medical mistrust [58–62, 92, 93]. This mistrust can influence healthcare-seeking behaviours, treatment adherence, and overall health outcomes [94, 95]. The intersectionality of race, gender, and sexual orientation creates unique challenges in healthcare with patients from racial minority groups experiencing higher levels of race-based and sexual/gender minority-based medical mistrust [58]. This dual burden of discrimination highlights the complex interplay between various aspects of identity and their impact on healthcare experiences. Female patients, for instance, often experience worse health outcomes due to compounded bias, further emphasising the multifaceted nature of healthcare disparities [43, 55]. The experiences of these individuals serve as a poignant example of how various forms of discrimination can intersect and amplify each other in healthcare settings, contributing to a cycle of mistrust and potentially poorer health outcomes.

Perceived discrimination in medical settings is significantly associated with decreased willingness to participate in clinical trials [59]. This is supported by White British individuals being 64% more likely to have participated in research and the continuous lack of representation of ethnic minorities in COVID- 19 research [96, 97]. By deterring participation in research, this perpetuates health disparities, potentially resulting in less effective treatments and interventions for these groups. This underrepresentation extends to medical literature and imagery, creating gaps in our understanding of how diseases and treatments affect different racial and ethnic groups [80–83]. This lack of diversity across various specialties can lead to delayed or missed diagnoses [25, 80–83] and reinforce existing stereotypes and bias among HPs.

Finally, this underrepresentation in research and literature circles back to perpetuate educational disparities. Healthcare education that lacks diverse representation in its materials and research base continues to produce HPs who may carry biases into their practice.

This self-sustaining cycle ensures the persistence and potential worsening of racial bias in healthcare and education, creating a continuous feedback loop where themes can also intersect at any points, reinforcing existing inequalities in the healthcare system. Addressing this complex issue requires a multifaceted approach, targeting each aspect of the cycle to break its perpetuation and promote equitable healthcare for all populations.

This systematic review highlights a gap in study specifically targeting methods to reduce racial bias in healthcare and education. Nonetheless, the insights gathered emphasise the need for comprehensive strategies to address this problem.

## Recommendations for Practice

To mitigate racial bias in healthcare practice, implementing thorough implicit bias training for HPs is crucial. This training should focus on accurate assessment and strategies to achieve equity in care for patients from diverse backgrounds [40, 42, 43, 48, 52, 53, 58, 73]. Standardising clinical processes and protocols, particularly in high-stakes areas like emergency departments and triage, can help reduce the impact of conscious and unconscious bias on patient access and care quality [5, 56]. Healthcare institutions should adopt multilevel strategies to build trust with minoritised populations by addressing both historical and ongoing discrimination [58]. Additionally, national guidelines should be updated to reflect individual patient needs, promoting a patient-centred approach rather than one guided by race [46, 57]. While not the only solution, increasing diversity in healthcare leadership and decision-making positions is also crucial to ensure diverse perspectives are represented in policy-making and organisational culture [79].

## Recommendations for Education

In healthcare education, incorporating equity, diversity, inclusion, and anti-racism as core competencies in curricula is essential. These topics should be integrated longitudinally and taught by educators with formal training and lived experiences of marginalisation [64]. Enhancing representation in educational materials, including textbooks and clinical images, can counteract implicit bias and improve care for patients with diverse skin tones and racial backgrounds [82]. Revising medical and healthcare education curricula to include comprehensive training on health disparities, social determinants of health, and the historical context of racism in healthcare, along with embedding cultural humility and structural competency training across medical, nursing, and allied health professional education through national mandates, can help reduce implicit bias and discrimination from the outset of healthcare professionals’ careers [42, 43, 61]. Educational organisations need to work collaboratively with all stakeholders to develop and implement consistent, evidence-based approaches to addressing racial bias in healthcare education [77].

## Recommendations for Research

Research recommendations include further studies on unconscious bias and provider clinical decision-making [49, 59, 68], considering the intersectional interplay of race, gender, and social determinants [55]. Future research should focus on evaluating the effectiveness of interventions aimed at reducing healthcare discrimination and their impact on racial and ethnic disparities in health outcomes [62, 75]. Additionally, effort in advancing evaluations of clinician-level interventions, such as implicit bias training, to address persistent inequities in access and outcomes should be made [43]. Increasing representation of minority researchers and participants in clinical research is crucial to ensure findings are applicable across diverse populations [59]. Implementing multifaceted anti-racist approaches in healthcare institutions and documenting their effectiveness can help adjust strategies to promote health equity [65].

Implementing these recommendations can help healthcare systems take significant steps towards breaking the cycle of racial bias and fostering a more equitable healthcare environment for all patients. Future directions should include promoting community-based participatory research approaches to ensure relevance, acceptability, and foster trust between researchers and affected communities.

The systematic review’s strengths include a robust methodology with comprehensive database searches, hand-searching specific journals, and interprofessional multi-reviewer processes that effectively mitigated individual bias and selection errors. The interprofessionality element of the team of researchers also added depth to the analysis. This diversity allowed for a more comprehensive understanding of the issues, as different perspectives were considered. Inductive thematic analysis enabled a nuanced interpretation of racial bias complexities in healthcare and education, enriching the key findings and conclusions.

However, the review’s limitations are intertwined with those of the included studies. The predominance of USA-based research may introduce geographical bias, potentially limiting its global applicability. Nevertheless, the topic of racial bias remains universally relevant. Although the manifestations and impact of racial bias can vary across different contexts, it persists as a critical factor of healthcare equity worldwide and in some instances echo national findings. Examples in the UK are highlighted by the Women and Equalities Committee [8], which reports that Black maternal mortality rates are almost four times higher than those for White women. Additionally, data from the National Child Mortality Database [9] indicates that the child death rate for ethnic minority children is more than double the rate of children from a White British ethnic background, mirroring similar disparities seen in the US.

Cross-sectional study prevalence limits causal inference, yet inclusion of longitudinal studies and large national datasets offers valuable insights into trends over time. The exclusion of studies with fewer than 100 participants improved reliability of effect size estimates and reduced publication bias, though it may have omitted relevant research, especially concerning rare conditions or novel interventions. The review did not extensively explore the intersectionality of race with other factors such as gender, socioeconomic status, and sexual orientation. Although the literature suggests that biases could impact workforce retention, direct correlations were not central in the topics analysed. Lastly, despite the inability to retrieve a large number of records, the focus on publicly accessible data supports transparency and reproducibility.

## Conclusions

This systematic review revealed that racial bias remains a pervasive issue in healthcare and healthcare education, manifesting across multiple interconnected domains. The identified themes—disparities in healthcare access and provision, perceived discrimination and medical mistrust, provider bias and stereotyping, disparities in healthcare education and training, and disparities in healthcare literature—form a self-reinforcing cycle that perpetuates racial inequities. Addressing these complex issues requires a multifaceted approach, including comprehensive implicit bias training, educational curricula revisions, standardisation of clinical processes, and increased diversity in healthcare leadership. Future research should focus on evaluating intervention effectiveness, advancing clinician-level interventions, and exploring intersectionality with other social determinants of health. Implementing these recommendations can help break the cycle of racial bias and foster a more equitable healthcare environment for all patients.

## Supplementary Information

Below is the link to the electronic supplementary material.

Supplementary file1 (DOCX 370 KB)

## Data Availability

This is a systematic review of relevant literature using the PRISM guidelines. No original data was collected for this study. All data utilised in this study has been incorporated into the manuscript and its supplementary materials.
