# Supplementary material for: The Perpetual Cycle of Racial Bias in Healthcare and Healthcare Education: A Systematic Review
Source: J Racial Ethn Health Disparities. 2025 Apr 16;13(3):2278–89. doi: 10.1007/s40615-025-02417-6 (PMC13157402; doi:10.1007/s40615-025-02417-6)
Supplement: Supplementary file 1 — Supplementary file1 (DOCX 370 KB) [file 40615_2025_2417_MOESM1_ESM.docx]

# **Supplementary Information**

## **Appendix I – Table 1 – Search Strategy Details**

| **PICo Framework Component** | **Description** |
| --- | --- |
| **Population** | Healthcare professionals, patients from diverse racial and ethnic backgrounds accessing healthcare, and students in healthcare-related studies. |
| **Interest/Phenomenon** | Racism and racist behaviours, implicit or explicit, in healthcare relationships (e.g., provider-patient, provider-provider, mentor-student, student-patient). Focus on interventions or strategies to mitigate racial bias. |
| **Context** | Any healthcare setting (short-term, long-term, acute care, physical and mental health services) and healthcare-related educational settings, including learning materials. |
| **Study Design** | Primary research studies, anticipating a focus on observational, qualitative, or mixed-methods studies, and less emphasis on randomized controlled trials. |
| **Key terms** | A combination of key terms and subject-headings were used relating to **bias** (“implicit bias” OR “implicit attitude” OR “implicit prejudice” OR “conscious bias” OR “conscious attitude” OR “conscious prejudice” OR “unconscious bias” OR “unconscious attitude” OR “unconscious prejudice”), **race/ethnicity** (race OR racial OR ethnic OR ethnicity OR minorit*), **healthcare** (“health” OR “healthcare” OR “health care”), and **healthcare-related education** (“health education”) and combined with Boolean operators “AND” and “OR”. The terms implicit and unconscious were selected as they emphasise the involuntary nature of these bias, underlining the importance of raising awareness while exploring strategies to mitigate their influence on healthcare and education. Conscious was included to ensure the search encompassed a comprehensive picture of the current racial bias-related healthcare panorama. |

## **Appendix II – Figure 1 – Conceptual Model: The Cyclical Nature of Racial Bias in Healthcare and Healthcare Education (HE)**


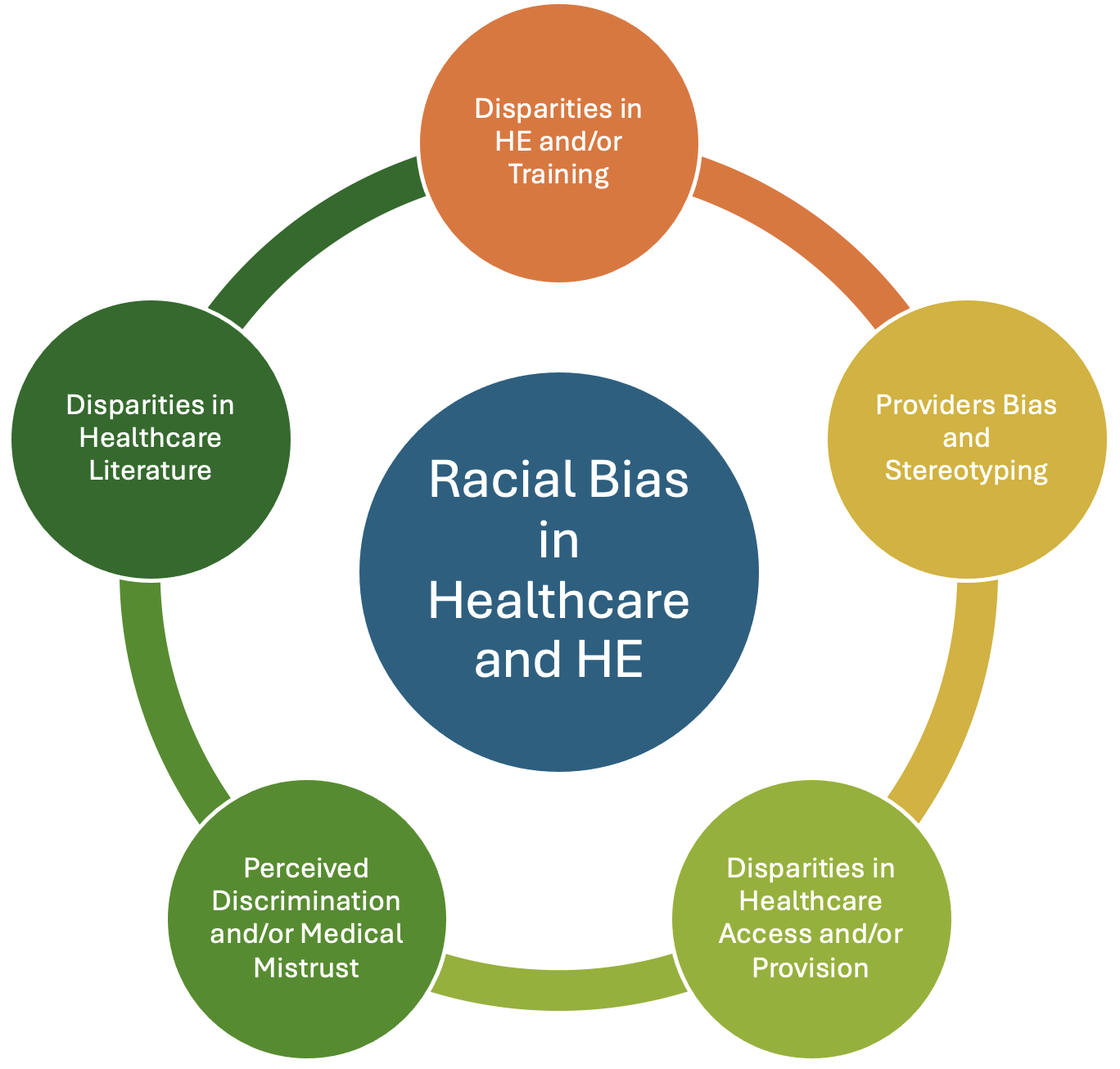


## **Appendix III – Figure 2 – PRISMA Flow Diagram^1^**


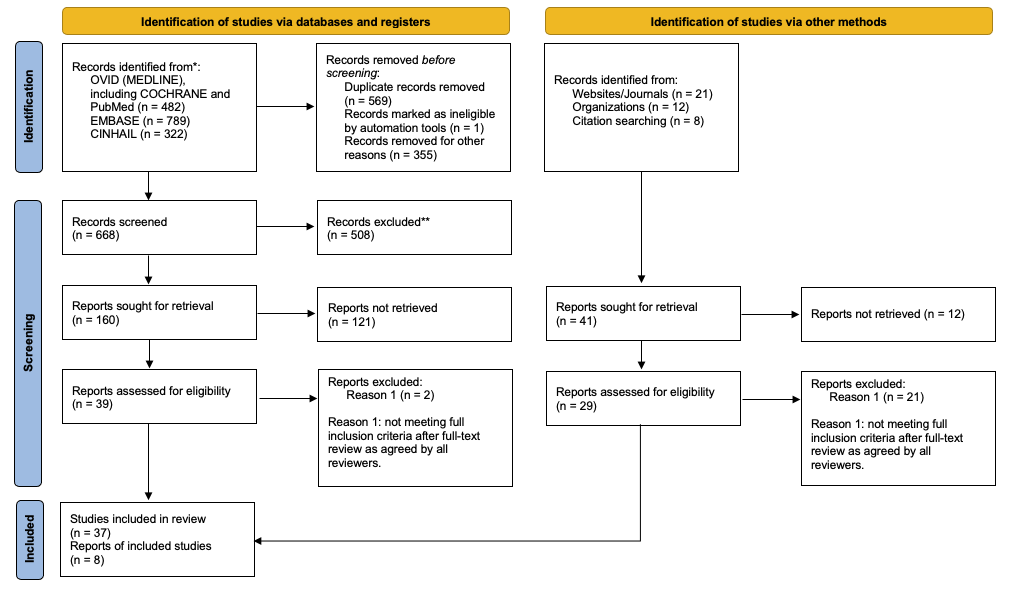


**References:**
1. Page MJ, McKenzie JE, Bossuyt PM, et al. The PRISMA 2020 statement: An updated guideline for reporting systematic reviews. *British Medical Journal*. 2021;372(n71). doi:https://doi.org/10.1136/bmj.n71

## **Appendix IV – Figure 3 – Distribution of Study Design per Geographical Location**

**Study Designs**

The included studies employed a range of research designs:

- Cross-sectional studies were the most common (n= 23) [1-23], allowing for examination of racial bias at a specific point in time across various healthcare settings and populations.
- Retrospective cohort studies (n=10) [24-33] analysed existing data to investigate racial disparities in healthcare outcomes and access over time.
- Prospective studies (n=3) [34-36] followed participants to assess the development or impact of racial bias longitudinally.
- Randomised studies (n=2) [37,38] used experimental designs to examine how race influences healthcare providers' perceptions and decision-making.
- Other designs included ecological studies, observational cohort, mixed methods approaches, and qualitative analyses (n=7) [39-45].

**Geographic Distribution**

Most studies were conducted in the United States of America (USA) (n=35) [1-3,5-13,15-20,22-24,26-30,32-35,38,39,41,42,45], reflecting a strong focus on racial bias in the American healthcare system. Only one study included global analysis of internation data [4], while other countries represented included:

- United Kingdom (UK) (n=5) [25,31,40,43,44], one of which specifically in England [44].
- Australia (n=1) [14].
- Canada (n=1) [21].
- Belgium (n=1) [36].
- Netherlands (n=1) [37].

**References:**

1. Walls ML, Gonzalez J, Gladney T, Onello E. Unconscious Biases: Racial Microaggressions in American Indian Health Care. *The Journal of the American Board of Family Medicine*. 2015;28(2):231-239. doi:https://doi.org/10.3122/jabfm.2015.02.140194

2. Puumala SE, Burgess KM, Kharbanda AB, et al. The Role of Bias by Emergency Department Providers in Care for American Indian Children. *Medical Care*. 2016;54(6):562-569. doi:https://doi.org/10.1097/mlr.0000000000000533

3. Hymel KP, Laskey AL, Crowell KR, et al. Racial and Ethnic Disparities and Bias in the Evaluation and Reporting of Abusive Head Trauma. *The Journal of Pediatrics*. 2018;198:137-143.e1. doi:https://doi.org/10.1016/j.jpeds.2018.01.048

4. Massie JP, Cho DY, Kneib CJ, et al. Patient Representation in Medical Literature: Are We Appropriately Depicting Diversity? *Plastic and Reconstructive Surgery – Global Open*. 2019;7(12):e2563. doi:https://doi.org/10.1097/GOX.0000000000002563

5. Devlin A, Gonzalez E, Ramsey F, Esnaola N, Fisher S. The Effect of Discrimination on Likelihood of Participation in a Clinical Trial. *Journal of racial and ethnic health disparities*. 2020;7(6):1124-1129. doi:https://doi.org/10.1007/s40615-020-00735-5

6. Hausmann LRM, Jones AL, McInnes SE, Zickmund SL. Identifying healthcare experiences associated with perceptions of racial/ethnic discrimination among veterans with pain: A cross-sectional mixed methods survey. Laws MB, ed. *PLOS ONE*. 2020;15(9):e0237650. doi:https://doi.org/10.1371/journal.pone.0237650

7. Holmes L, O’Neill L, Elmi H, et al. Implication of Vaginal and Cesarean Section Delivery Method in Black–White Differentials in Infant Mortality in the United States: Linked Birth/Infant Death Records, 2007–2016. *International Journal of Environmental Research and Public Health*. 2020;17(9):3146. doi:https://doi.org/10.3390/ijerph17093146

8. Johnson-Jennings M, Duran B, Hakes J, Paffrath A, Little MM. The influence of undertreated chronic pain in a national survey: Prescription medication misuse among American indians, Asian Pacific Islanders, Blacks, Hispanics and whites. *SSM - Population Health*. 2020;11:100563. doi:https://doi.org/10.1016/j.ssmph.2020.100563

9. Pro G, Zaller N. Interaction effects in the association between methadone maintenance therapy and experiences of racial discrimination in U.S. healthcare settings. *PLOS ONE*. 2020;15(2):e0228755. doi:https://doi.org/10.1371/journal.pone.0228755

10. Agarwal S, Schechter C, Gonzalez J, Long JA. Racial-Ethnic Disparities in Diabetes Technology Use Among Young Adults with Type 1 Diabetes. *Diabetes Technology & Therapeutics*. 2021;23(4):306-313. doi:https://doi.org/10.1089/dia.2020.0338

11. Basile Ibrahim B, Kennedy HP, Combellick J. Experiences of Quality Perinatal Care During the US COVID‐19 Pandemic. *Journal of Midwifery & Women’s Health*. 2021;66(5):579-588. doi:https://doi.org/10.1111/jmwh.13269

12. Bell G, Holmes S, Gillespie S, Wood A, Murray BL. Images of dark skin in top emergency medicine journals: A cross‐sectional analysis of images of emergent cutaneous disorders. *AEM Education and Training*. 2021;5(S1):S76-S81. doi:https://doi.org/10.1002/aet2.10683

13. Rana A, Witt A, Jones H, Mwanthi M, Murray J, Zickuhr L. The Representation of Skin Colors in Images of Patients with Lupus Erythematosus. *Arthritis Care & Research*. 2021;74(11):1835-1841. doi:https://doi.org/10.1002/acr.24712

14. D’Costa I, Hunt I, Russell L, Adams K. A racial bias test with tertiary cancer centre employees: why anti-racist measures are required for First Nations Australians cancer care equity. *Australian Health Review*. 2022;47(1):5-12. doi:https://doi.org/10.1071/AH21113

15. Hagiwara N, Duffy C, Quillin J. Implicit and explicit racial prejudice and stereotyping toward Black (vs. White) Americans: The prevalence and variation among genetic counselors in North America. *Journal of Genetic Counseling*. 2022;32(2):397-410. doi:https://doi.org/10.1002/jgc4.1648

16. Javed Z, Maqsood MH, Amin Z, Nasir K. Race and Ethnicity and Cardiometabolic Risk Profile: Disparities Across Income and Health Insurance in a National Sample of US Adults. *Journal of Public Health Management and Practice*. 2022;28(1):S91-S100. doi:https://doi.org/10.1097/phh.0000000000001441

17. Kalantari A, Alvarez A, Chung A, et al. Sex and Race Visual Representation in Emergency Medicine Textbooks and the Hidden Curriculum. *AEM Education and Training*. 2022;6(3). doi:https://doi.org/10.1002/aet2.10743

18. Cox AB, Jaiswal J, LoSchiavo C, et al. Medical Mistrust Among a Racially and Ethnically Diverse Sample of Sexual Minority Men. *LGBT health*. 2023;10(6):471-479. doi:https://doi.org/10.1089/lgbt.2022.0252

19. Green TL, Vu H, Laura E.T. Swan, et al. Implicit and explicit racial prejudice among medical professionals: updated estimates from a population-based study. *The Lancet Regional Health - Americas*. 2023;21:100489-100489. doi:https://doi.org/10.1016/j.lana.2023.100489

20. Huang BB, Saseendrakumar BR, Delavar AG, Baxter SL. Racial Disparities in Barriers to Care for Patients With Diabetic Retinopathy in a Nationwide Cohort. *Translational Vision Science & Technology*. 2023;12(3):14-14. doi:https://doi.org/10.1167/tvst.12.3.14

21. Roach P, Ruzycki SM, Hernandez S, et al. Prevalence and characteristics of anti-Indigenous bias among Albertan physicians: a cross-sectional survey and framework analysis. *BMJ Open*. 2023;13(2):e063178. doi:https://doi.org/10.1136/bmjopen-2022-063178

22. Sangal RB, Su H, Khidir H, et al. Sociodemographic Disparities in Queue Jumping for Emergency Department Care. *JAMA Network Open*. 2023;6(7):e2326338. doi:https://doi.org/10.1001/jamanetworkopen.2023.26338

23. Won Yoo J, Kang HT, Choe I, et al. Racial and Ethnic Disparity in 4Ms among Older Adults Among Telehealth Users as Primary Care. *Gerontology & geriatric medicine*. 2023;9:1-8. doi:https://doi.org/10.1177/23337214231189053

24. Ali I, Vattigunta S, Jang JM, et al. Racial Disparities are Present in the Timing of Radiographic Assessment and Surgical Treatment of Hip Fractures. *Clinical Orthopaedics and Related Research*. 2020;478(3):455-461. doi:https://doi.org/10.1097/CORR.0000000000001091

25. Sinnott SJ, Douglas IJ, Smeeth L, Williamson E, Tomlinson LA. First line drug treatment for hypertension and reductions in blood pressure according to age and ethnicity: cohort study in UK primary care. *BMJ*. 2020;371(8269). doi:https://doi.org/10.1136/bmj.m4080

26. Amdani S, Bhimani SA, Boyle G, et al. Racial and Ethnic Disparities Persist in the Current Era of Pediatric Heart Transplantation. *Journal of Cardiac Failure*. 2021;27(9):957-964. doi:https://doi.org/10.1016/j.cardfail.2021.05.027

27. Cascino T, Somanchi S, Colvin M, et al. Racial and Sex Inequities in the Use of and Outcomes After Left Ventricular Assist Device Implantation Among Medicare Beneficiaries. *JAMA network open*. 2022;5(7):e2223080. doi:https://doi.org/10.1001/jamanetworkopen.2022.23080

28. Holt HK, Gildengorin G, Karliner L, Fontil V, Pramanik R, Potter MB. Differences in Hypertension Medication Prescribing for Black Americans and Their Association with Hypertension Outcomes. *The Journal of the American Board of Family Medicine*. 2022;35(1):26-34. doi:https://doi.org/10.3122/jabfm.2022.01.210276

29. Liu TL, Taylor YJ, Schuch JC, Tucker L, Zager KM, Dulin MF. Variations in Receipt of Contraceptives by Insurance Status and Race/Ethnicity. *North Carolina Medical Journal*. 2022;83(1):58-66. doi:https://doi.org/10.18043/ncm.83.1.58

30. Longcoy J, Patwari R, Hasler S, et al. Racial and Ethnic Differences in Hospital Admissions of Emergency Department COVID-19 Patients. *Medical Care*. 2022;60(6):415-422. doi:https://doi.org/10.1097/mlr.0000000000001710

31. Brown C, Goss C, Sam AH. Is the awarding gap at UK medical schools influenced by ethnicity and medical school attended? A retrospective cohort study. *BMJ Open*. 2023;13(12):e075945. doi:https://doi.org/10.1136/bmjopen-2023-075945

32. Pacheco F, Luciano E, Hebert D, Serpa E, Solh W. Does race influence the attainment of the principles of oncologic surgical resection in colon adenocarcinoma? A Retrospective cohort analysis from the National Cancer Database. *Annals of Medicine and Surgery*. 2023;85(5):1562-1565. doi:https://doi.org/10.1097/ms9.0000000000000693

33. Topaz M, Song J, Davoudi A, et al. Home Health Care Clinicians’ Use of Judgment Language for Black and Hispanic Patients: Natural Language Processing Study. *JMIR nursing*. 2023;6:e42552. doi:https://doi.org/10.2196/42552

34. Blair IV, Steiner JF, Hanratty R, et al. An Investigation of Associations Between Clinicians’ Ethnic or Racial Bias and Hypertension Treatment, Medication Adherence and Blood Pressure Control. *Journal of General Internal Medicine*. 2014;29(7):987-995. doi:https://doi.org/10.1007/s11606-014-2795-z

35. van Ryn M, Hardeman R, Phelan SM, et al. Medical School Experiences Associated with Change in Implicit Racial Bias Among 3547 Students: A Medical Student CHANGES Study Report. *Journal of General Internal Medicine*. 2015;30(12):1748-1756. doi:https://doi.org/10.1007/s11606-015-3447-7

36. Duveau C, Demoulin S, Dauvrin M, Lepièce B, Lorant V. Implicit and explicit ethnic biases in multicultural primary care: the case of trainee general practitioners. *BMC Primary Care*. 2022;23(1). doi:https://doi.org/10.1186/s12875-022-01698-8

37. van Andel CEE, Born MP, van den Broek WW, Stegers‐Jager KM. Do norms unintentionally increase stereotypical expressions? A randomised controlled trial. *Medical Education*. 2021;56(3):331-338. doi:https://doi.org/10.1111/medu.14712

38. Bunting SR, Feinstein BA, Calabrese SK, et al. Assumptions about patients seeking PrEP: Exploring the effects of patient and sexual partner race and gender identity and the moderating role of implicit racism. *PLOS ONE*. 2022;17(7):e0270861. doi:https://doi.org/10.1371/journal.pone.0270861

39. Burton É, Flores B, Jerome B, et al. Assessment of Bias in Patient Safety Reporting Systems Categorized by Physician Gender, Race and Ethnicity, and Faculty Rank. *JAMA Network Open*. 2022;5(5):e2213234. doi:https://doi.org/10.1001/jamanetworkopen.2022.13234

40. Mukherji P, Adas MA, Clarke B, et al. Changing trends in ethnicity and academic performance: observational cohort data from a UK medical school. *BMJ Open*. 2022;12(12):e066886. doi:https://doi.org/10.1136/bmjopen-2022-066886

41. Nikpour J, Broome M, Silva S, Allen KD. Patient demographics and clinical characteristics influence opioid and nonopioid pain management prescriptions of primary care NPs, PAs, and physicians. *Journal of the American Association of Nurse Practitioners*. 2022;34(7):883-890. doi:https://doi.org/10.1097/jxx.0000000000000728

42. Rambachan A, Abe-Jones Y, Fernandez A, Shahram Y. Racial Disparities in 7-Day Readmissions from an Adult Hospital Medicine Service. *Journal of Racial and Ethnic Health Disparities*. 2022;9(4):1500-1505. doi:https://doi.org/10.1007/s40615-021-01088-3

43. Pinder RJ, Bury F, Sathyamoorthy G, Majeed A, Rao M. Differential attainment in specialty training recruitment in the UK: an observational analysis of the impact of psychometric testing assessment in Public Health postgraduate selection. *BMJ Open*. 2023;13(3):e069738-e069738. doi:https://doi.org/10.1136/bmjopen-2022-069738

44. Rice CT, Barnett S, O’Connell SP, et al. Impact of gender, ethnicity and social deprivation on access to surgical or transcatheter aortic valve replacement in aortic stenosis: a retrospective database study in England. *Open Heart*. 2023;10(2):e002373. doi:https://doi.org/10.1136/openhrt-2023-002373

45. Holmes L, Enwere M, Mason R, et al. Medical Misadventures as Errors and Mistakes and Motor Vehicular Accidents in the Disproportionate Burden of Childhood Mortality among Blacks/African Americans in the United States: CDC Dataset, 1968–2015. *Healthcare*. 2024;12(4):477-477. doi:https://doi.org/10.3390/healthcare12040477

## **Appendix V – Figure 4 – Study Population Categories by Included Study**

**Study Population**

The studies included the following population categories:

- Patients only (n=26) [1-26]: These studies focused on racial disparities in healthcare access, treatment, and outcomes among diverse patient groups.
- Healthcare professionals only (n=5) [27-31]: These studies examined racial bias among physicians, nurses, and other healthcare providers.
- Both patients and healthcare professionals (n=3) [32-34]: These studies investigated the interaction between provider bias and patient experiences or outcomes.
- Healthcare students only (n=6) [35-40], one of which also included teachers as an ecological valid choice [40]: These studies focused on racial bias in medical education and among trainees.
- Both healthcare students and professionals (n=1) [41]: This study evaluated the presence of racial bias amongst both certified professionals and genetic counsellors in training.
- Indirect patient and/or healthcare professional representation (n=4) [42-45]: These studies analysed racial representation in medical literature and imaging.

**References:**

1. Walls ML, Gonzalez J, Gladney T, Onello E. Unconscious Biases: Racial Microaggressions in American Indian Health Care. *The Journal of the American Board of Family Medicine*. 2015;28(2):231-239. doi:https://doi.org/10.3122/jabfm.2015.02.140194

2. Hymel KP, Laskey AL, Crowell KR, et al. Racial and Ethnic Disparities and Bias in the Evaluation and Reporting of Abusive Head Trauma. *The Journal of Pediatrics*. 2018;198:137-143.e1. doi:https://doi.org/10.1016/j.jpeds.2018.01.048

3. Ali I, Vattigunta S, Jang JM, et al. Racial Disparities are Present in the Timing of Radiographic Assessment and Surgical Treatment of Hip Fractures. *Clinical Orthopaedics and Related Research*. 2020;478(3):455-461. doi:https://doi.org/10.1097/CORR.0000000000001091

4. Devlin A, Gonzalez E, Ramsey F, Esnaola N, Fisher S. The Effect of Discrimination on Likelihood of Participation in a Clinical Trial. *Journal of racial and ethnic health disparities*. 2020;7(6):1124-1129. doi:https://doi.org/10.1007/s40615-020-00735-5

5. Hausmann LRM, Jones AL, McInnes SE, Zickmund SL. Identifying healthcare experiences associated with perceptions of racial/ethnic discrimination among veterans with pain: A cross-sectional mixed methods survey. Laws MB, ed. *PLOS ONE*. 2020;15(9):e0237650. doi:https://doi.org/10.1371/journal.pone.0237650

6. Holmes L, O’Neill L, Elmi H, et al. Implication of Vaginal and Cesarean Section Delivery Method in Black–White Differentials in Infant Mortality in the United States: Linked Birth/Infant Death Records, 2007–2016. *International Journal of Environmental Research and Public Health*. 2020;17(9):3146. doi:https://doi.org/10.3390/ijerph17093146

7. Johnson-Jennings M, Duran B, Hakes J, Paffrath A, Little MM. The influence of undertreated chronic pain in a national survey: Prescription medication misuse among American indians, Asian Pacific Islanders, Blacks, Hispanics and whites. *SSM - Population Health*. 2020;11:100563. doi:https://doi.org/10.1016/j.ssmph.2020.100563

8. Pro G, Zaller N. Interaction effects in the association between methadone maintenance therapy and experiences of racial discrimination in U.S. healthcare settings. *PLOS ONE*. 2020;15(2):e0228755. doi:https://doi.org/10.1371/journal.pone.0228755

9. Sinnott SJ, Douglas IJ, Smeeth L, Williamson E, Tomlinson LA. First line drug treatment for hypertension and reductions in blood pressure according to age and ethnicity: cohort study in UK primary care. *BMJ*. 2020;371(8269). doi:https://doi.org/10.1136/bmj.m4080

10. Agarwal S, Schechter C, Gonzalez J, Long JA. Racial-Ethnic Disparities in Diabetes Technology Use Among Young Adults with Type 1 Diabetes. *Diabetes Technology & Therapeutics*. 2021;23(4):306-313. doi:https://doi.org/10.1089/dia.2020.0338

11. Amdani S, Bhimani SA, Boyle G, et al. Racial and Ethnic Disparities Persist in the Current Era of Pediatric Heart Transplantation. *Journal of Cardiac Failure*. 2021;27(9):957-964. doi:https://doi.org/10.1016/j.cardfail.2021.05.027

12. Basile Ibrahim B, Kennedy HP, Combellick J. Experiences of Quality Perinatal Care During the US COVID‐19 Pandemic. *Journal of Midwifery & Women’s Health*. 2021;66(5):579-588. doi:https://doi.org/10.1111/jmwh.13269

13. Cascino T, Somanchi S, Colvin M, et al. Racial and Sex Inequities in the Use of and Outcomes After Left Ventricular Assist Device Implantation Among Medicare Beneficiaries. *JAMA network open*. 2022;5(7):e2223080. doi:https://doi.org/10.1001/jamanetworkopen.2022.23080

14. Holt HK, Gildengorin G, Karliner L, Fontil V, Pramanik R, Potter MB. Differences in Hypertension Medication Prescribing for Black Americans and Their Association with Hypertension Outcomes. *The Journal of the American Board of Family Medicine*. 2022;35(1):26-34. doi:https://doi.org/10.3122/jabfm.2022.01.210276

15. Javed Z, Maqsood MH, Amin Z, Nasir K. Race and Ethnicity and Cardiometabolic Risk Profile: Disparities Across Income and Health Insurance in a National Sample of US Adults. *Journal of Public Health Management and Practice*. 2022;28(1):S91-S100. doi:https://doi.org/10.1097/phh.0000000000001441

16. Liu TL, Taylor YJ, Schuch JC, Tucker L, Zager KM, Dulin MF. Variations in Receipt of Contraceptives by Insurance Status and Race/Ethnicity. *North Carolina Medical Journal*. 2022;83(1):58-66. doi:https://doi.org/10.18043/ncm.83.1.58

17. Longcoy J, Patwari R, Hasler S, et al. Racial and Ethnic Differences in Hospital Admissions of Emergency Department COVID-19 Patients. *Medical Care*. 2022;60(6):415-422. doi:https://doi.org/10.1097/mlr.0000000000001710

18. Nikpour J, Broome M, Silva S, Allen KD. Patient demographics and clinical characteristics influence opioid and nonopioid pain management prescriptions of primary care NPs, PAs, and physicians. *Journal of the American Association of Nurse Practitioners*. 2022;34(7):883-890. doi:https://doi.org/10.1097/jxx.0000000000000728

19. Rambachan A, Abe-Jones Y, Fernandez A, Shahram Y. Racial Disparities in 7-Day Readmissions from an Adult Hospital Medicine Service. *Journal of Racial and Ethnic Health Disparities*. 2022;9(4):1500-1505. doi:https://doi.org/10.1007/s40615-021-01088-3

20. Cox AB, Jaiswal J, LoSchiavo C, et al. Medical Mistrust Among a Racially and Ethnically Diverse Sample of Sexual Minority Men. *LGBT health*. 2023;10(6):471-479. doi:https://doi.org/10.1089/lgbt.2022.0252

21. Huang BB, Saseendrakumar BR, Delavar AG, Baxter SL. Racial Disparities in Barriers to Care for Patients With Diabetic Retinopathy in a Nationwide Cohort. *Translational Vision Science & Technology*. 2023;12(3):14-14. doi:https://doi.org/10.1167/tvst.12.3.14

22. Pacheco F, Luciano E, Hebert D, Serpa E, Solh W. Does race influence the attainment of the principles of oncologic surgical resection in colon adenocarcinoma? A Retrospective cohort analysis from the National Cancer Database. *Annals of Medicine and Surgery*. 2023;85(5):1562-1565. doi:https://doi.org/10.1097/ms9.0000000000000693

23. Rice CT, Barnett S, O’Connell SP, et al. Impact of gender, ethnicity and social deprivation on access to surgical or transcatheter aortic valve replacement in aortic stenosis: a retrospective database study in England. *Open Heart*. 2023;10(2):e002373. doi:https://doi.org/10.1136/openhrt-2023-002373

24. Sangal RB, Su H, Khidir H, et al. Sociodemographic Disparities in Queue Jumping for Emergency Department Care. *JAMA Network Open*. 2023;6(7):e2326338. doi:https://doi.org/10.1001/jamanetworkopen.2023.26338

25. Won Yoo J, Kang HT, Choe I, et al. Racial and Ethnic Disparity in 4Ms among Older Adults Among Telehealth Users as Primary Care. *Gerontology & geriatric medicine*. 2023;9:1-8. doi:https://doi.org/10.1177/23337214231189053

26. Holmes L, Enwere M, Mason R, et al. Medical Misadventures as Errors and Mistakes and Motor Vehicular Accidents in the Disproportionate Burden of Childhood Mortality among Blacks/African Americans in the United States: CDC Dataset, 1968–2015. *Healthcare*. 2024;12(4):477-477. doi:https://doi.org/10.3390/healthcare12040477

27. Puumala SE, Burgess KM, Kharbanda AB, et al. The Role of Bias by Emergency Department Providers in Care for American Indian Children. *Medical Care*. 2016;54(6):562-569. doi:https://doi.org/10.1097/mlr.0000000000000533

28. Burton É, Flores B, Jerome B, et al. Assessment of Bias in Patient Safety Reporting Systems Categorized by Physician Gender, Race and Ethnicity, and Faculty Rank. *JAMA Network Open*. 2022;5(5):e2213234. doi:https://doi.org/10.1001/jamanetworkopen.2022.13234

29. D’Costa I, Hunt I, Russell L, Adams K. A racial bias test with tertiary cancer centre employees: why anti-racist measures are required for First Nations Australians cancer care equity. *Australian Health Review*. 2022;47(1):5-12. doi:https://doi.org/10.1071/AH21113

30. Pinder RJ, Bury F, Sathyamoorthy G, Majeed A, Rao M. Differential attainment in specialty training recruitment in the UK: an observational analysis of the impact of psychometric testing assessment in Public Health postgraduate selection. *BMJ Open*. 2023;13(3):e069738-e069738. doi:https://doi.org/10.1136/bmjopen-2022-069738

31. Roach P, Ruzycki SM, Hernandez S, et al. Prevalence and characteristics of anti-Indigenous bias among Albertan physicians: a cross-sectional survey and framework analysis. *BMJ Open*. 2023;13(2):e063178. doi:https://doi.org/10.1136/bmjopen-2022-063178

32. Blair IV, Steiner JF, Hanratty R, et al. An Investigation of Associations Between Clinicians’ Ethnic or Racial Bias and Hypertension Treatment, Medication Adherence and Blood Pressure Control. *Journal of General Internal Medicine*. 2014;29(7):987-995. doi:https://doi.org/10.1007/s11606-014-2795-z

33. Green TL, Vu H, Laura E.T. Swan, et al. Implicit and explicit racial prejudice among medical professionals: updated estimates from a population-based study. *The Lancet Regional Health - Americas*. 2023;21:100489-100489. doi:https://doi.org/10.1016/j.lana.2023.100489

34. Topaz M, Song J, Davoudi A, et al. Home Health Care Clinicians’ Use of Judgment Language for Black and Hispanic Patients: Natural Language Processing Study. *JMIR nursing*. 2023;6:e42552. doi:https://doi.org/10.2196/42552

35. van Ryn M, Hardeman R, Phelan SM, et al. Medical School Experiences Associated with Change in Implicit Racial Bias Among 3547 Students: A Medical Student CHANGES Study Report. *Journal of General Internal Medicine*. 2015;30(12):1748-1756. doi:https://doi.org/10.1007/s11606-015-3447-7

36. Bunting SR, Feinstein BA, Calabrese SK, et al. Assumptions about patients seeking PrEP: Exploring the effects of patient and sexual partner race and gender identity and the moderating role of implicit racism. *PLOS ONE*. 2022;17(7):e0270861. doi:https://doi.org/10.1371/journal.pone.0270861

37. Duveau C, Demoulin S, Dauvrin M, Lepièce B, Lorant V. Implicit and explicit ethnic biases in multicultural primary care: the case of trainee general practitioners. *BMC Primary Care*. 2022;23(1). doi:https://doi.org/10.1186/s12875-022-01698-8

38. Mukherji P, Adas MA, Clarke B, et al. Changing trends in ethnicity and academic performance: observational cohort data from a UK medical school. *BMJ Open*. 2022;12(12):e066886. doi:https://doi.org/10.1136/bmjopen-2022-066886

39. Brown C, Goss C, Sam AH. Is the awarding gap at UK medical schools influenced by ethnicity and medical school attended? A retrospective cohort study. *BMJ Open*. 2023;13(12):e075945. doi:https://doi.org/10.1136/bmjopen-2023-075945

40. van Andel CEE, Born MP, van den Broek WW, Stegers‐Jager KM. Do norms unintentionally increase stereotypical expressions? A randomised controlled trial. *Medical Education*. 2021;56(3):331-338. doi:https://doi.org/10.1111/medu.14712

41. Hagiwara N, Duffy C, Quillin J. Implicit and explicit racial prejudice and stereotyping toward Black (vs. White) Americans: The prevalence and variation among genetic counselors in North America. *Journal of Genetic Counseling*. 2022;32(2):397-410. doi:https://doi.org/10.1002/jgc4.1648

42. Massie JP, Cho DY, Kneib CJ, et al. Patient Representation in Medical Literature: Are We Appropriately Depicting Diversity? *Plastic and Reconstructive Surgery – Global Open*. 2019;7(12):e2563. doi:https://doi.org/10.1097/GOX.0000000000002563

43. Bell G, Holmes S, Gillespie S, Wood A, Murray BL. Images of dark skin in top emergency medicine journals: A cross‐sectional analysis of images of emergent cutaneous disorders. *AEM Education and Training*. 2021;5(S1):S76-S81. doi:https://doi.org/10.1002/aet2.10683

44. Rana A, Witt A, Jones H, Mwanthi M, Murray J, Zickuhr L. The Representation of Skin Colors in Images of Patients with Lupus Erythematosus. *Arthritis Care & Research*. 2021;74(11):1835-1841. doi:https://doi.org/10.1002/acr.24712

45. Kalantari A, Alvarez A, Chung A, et al. Sex and Race Visual Representation in Emergency Medicine Textbooks and the Hidden Curriculum. *AEM Education and Training*. 2022;6(3). doi:https://doi.org/10.1002/aet2.10743

## **Appendix VI – Figure 5 – Ethnic/Racial Groups Represented in Study Populations**

Please note that this table does not show percentages or exact numbers of participants from each ethnic/racial group per included study. Instead, it indicates the presence or absence of each group within the different population types across all included studies. This approach was chosen because not all studies under review reported detailed demographic breakdowns or percentages for each ethnic/racial group. A "1" in a cell should be interpreted as "at least one study included this ethnic/racial group in this population type," rather than as a count or percentage. This table aims to provide a broad overview of ethnic/racial representation in the reviewed studies, highlighting which groups were included in various population types, without making claims about the extent of that representation.

## **Appendix VII – Figure 6 – Distribution of Studies by Theme**

## **Appendix IX – Table 2 – Example of Data Extraction Table**

The data extraction form included the following fields:

| Field | Description |
| --- | --- |
| Study ID | RefWorks unique identifier number |
| Citation | Citation to include lead authors (up to three) and year of publication |
| Purpose/Aim/Objective | Concisely describe the study’s aim, purpose, or primary research question. |
| Study Design | Specify the specific design of the study (e.g., Randomised Controlled Trial, Cross-sectional study) |
| Location | Specify Geographical Location |
| Start and End Date | Indicate the timeframe of the study (from data collection to publication, if available). |
| Sampling Strategy | Describe how participants were selected (random, convenience, snowball sampling, etc.). |
| Sample Size | \|  \| \| --- \|  \| Provide the total number of participants in the study. \| \| --- \| |
| Population included   - Healthcare Professionals | If yes, specify the profession(s) and include race and ethnicity breakdown (percentages if provided). |
| Population included   - Healthcare Students | If yes, specify the course, year of study, and include race and ethnicity breakdown (percentages if provided). |
| Population included   - Patients | If yes, include race and ethnicity breakdown (percentages if provided). |
| Method used to assess potential racial bias | Explain the method(s) used to evaluate racial bias (e.g., specific validated tool, surveys, interviews, statistical analysis, implicit bias tests). |
| Clinical Area | Specify the healthcare field or specialty involved (e.g., oncology, cardiology, primary care, etc.). |
| Were Racial Biases Identified? | - Yes - No |
| Main Findings | Summarise the key findings of the study, especially regarding racial bias and any healthcare implications. |
| Correlation with Healthcare Outcomes Identified? | - Yes - No |
| Strengths of the Study | List the strengths such as rigorous methodology, large sample size, diversity of the population, etc. |
| Limitations of the Study | Outline the limitations (e.g., small sample size, limited geographic scope, selection bias). |
| Main Theme Extrapolated | What key theme or conclusion is drawn from the study? |
| Additional Notes | Space for any other relevant details or comments. |

## **Appendix X – Table 3 – Study Characteristics and Main Themes**

| **Citation** | **Design** | **Location** | **Population** | **Method** | **Main Findings** |  |
| --- | --- | --- | --- | --- | --- | --- |
| **Disparities in Healthcare Access and/or Provision** | | | | | | |
| Holmes et al. [1] | Retrospective Ecological Study | USA | Patients only: Black/African American (AA), White | No specific tool – assumption from mortality rate ratio and trend analysis, percent change calculation, and statistical significance testing. | Significant racial bias in medical care of Black AA infants evidenced by increased mortality disparities from medical errors over time, unlike traffic accident mortality. |  |
| Rice et al. [2] | Retrospective Observational Study | England | Patients only: White, South Asian, Black, Mixed, Other, Unknown | No specific tool – assumption from analysis of the odds of receiving Aortic Valve Replacement (AVR) among people with aortic stenosis based on gender, ethnicity and social deprivation status. | Female gender, Black or South Asian ethnicities, and high deprivation associated with reduced odds of receiving AVR in England, with a lower proportion of timely procedures for minority ethnicities and deprived individuals. |  |
| Sangal et al. [3] | Retrospective Cross-Sectional Study | USA | Patients only: Non-Hispanic White (NHW) (42.2%), Non-Hispanic Black (NHB) (33.8%), Hispanic/Latino (21.1%) | No specific tool – assumption from analysis of prevalence of Unexplained Queue Jumps (UQJ) and association with social factors, including race. | Marginalised populations more likely to experience UQJ, hallway placement, and leaving without treatment despite similar triage acuity levels, highlighting consistent disparities. |  |
| Pacheco et al. [4] | Retrospective Cohort Study | USA | Patients only: White, AA, Asian, Native American | No specific tool – assumption from multivariable logistic regression adjusted for demographic variables, including race. | Racial disparities in achieving adequate oncologic resection in colon cancer treatment, with AA and Native American patients less likely to achieve adequate resection compared to White patients. |  |
| Johnson-Jennings et al. [5] | Cross-Sectional Study | USA | Patients only: White (20,000), American Indian (AI)/Alaska Native (AN) (700), Black (6,400), Asian/Pacific Islanders (1,000), Hispanic (6,400) | No specific tool – assumption from analysis exploring relationship between pain intensity and Prescription Drug Misuse (PDM) rates across racial/ethnic groups, adjusting for health behaviour model factors. | Pain intensity's curvilinear relationship with PDM rates varied among racial/ethnic groups, suggesting undertreated chronic pain may affect outcomes differently across groups. |  |
| Huang et al. [6] | Cross-Sectional Study | USA | Patients only: NHB, Hispanic, and NHW | No specific tool – assumption from multivariable logistic regression models used to assess association between race/ethnicity and barriers to care. | NHB and Hispanic patients with diabetic retinopathy reported greater delays/barriers to care compared to NHW patients, irrespective of socioeconomic factors. |  |
| Cascino et al. [7] | Retrospective Cohort Study | USA | Patients only: Black (22.9%), White (77.1%) – Female sex (23.7%), Female Black (36.2%), Female White (20%) | No specific tool – assumption from analysis to assess racial and sex bias’ influence on Left Ventricular Assist Device (LVAD) use. | Racial disparities in receiving LVADs, with Black beneficiaries and female patients less likely to receive this therapy, indicating possible clinician decision-making differences. |  |
| Longcoy et al. [8] | Retrospective Cohort Study | USA | Patients only: White (16.1%), Black (40.1%), Hispanic (43.8%) | No specific tool – assumption from analysis of race/ethnicity adjusted association with Emergency Department (ED) discharge disposition and Intensive Care Unit (ICU) admission. | White patients had higher odds of hospital and ICU admissions from the ED for COVID-19 compared to Black patients, with no significant difference in hospital admissions between White and Hispanic patients. |  |
| Liu et al. [9] | Retrospective Cohort Study | USA | Patients only: NHB (32.2%), Hispanic (11.1%), NHW (45.8%), Other (10.9%) | No specific tool – assumption from analysis of race/ethnicity and socioeconomic factors. | NHB and Hispanic women with Medicaid had higher odds of receiving Long-Acting Reversible Contraceptives compared to NHW women with commercial insurance, highlighting disparities in contraceptive care. |  |
| Javed et al. [10] | Cross-Sectional Study | USA | Patients only: NHW (70%), NHB (13%), Hispanic (18%) | No specific tool – assumption from analysis of race and ethnicity’s association with Cardio-Metabolic Burden (CMB) profile by income and insurance type. | Racial disparities in CMB risk profiles persisted across income levels and insurance types, with NHB and Hispanics experiencing poorer outcomes. |  |
| Basile Ibrahim, Kennedy and Combellick [11] | Cross-Sectional, Explanatory Sequantial Mixed-Method Study | USA | Patients only: White (86.2%), Black, Indigenous, and People of Colour (BIPOC) (17.1%) | No specific tool – assumption from analysis of racial/ethnic identity on perinatal care quality. | Autonomy in perinatal care decision-making decreased during COVID-19, particularly affecting BIPOC, individuals with an obstetrician provider, and hospital births, indicating disparities in care quality. |  |
| Agarwal et al. [12] | Cross-Sectional Study | USA | Patients only: NHW (100), NHB (97), Hispanic (103) | No specific tool – assumption from impact analysis of race/ethnicity on diabetes technology use, adjusted for socioeconomic status, healthcare factors, and diabetes self-management. | Significant racial-ethnic disparities in insulin pump and continuous glucose monitor use among young adults with Type 1 Diabetes, not fully explained by socioeconomic status or diabetes management. |  |
| Ali et al. [13] | Retrospective Cohort Study | USA | Patients only: White (84%), Black (7.6%), Asian (4.5%), Other (3.9%) | No specific tool – assumption from review assessing associations between race and outcomes of interest. | Black patients experienced longer wait times for hip fracture evaluation and surgery compared to White patients, influenced by hospital type. |  |
| Amdani et al. [14] | Retrospective Cohort Study | USA | Patients only: Caucasian (52.2%), AA (20.5%), Hispanic (19.8%), Asian (3.9%), Other races (3.6%) | No specific tool – assumption from comparison of clinical characteristics and survival among children of different racial/ethnic groups at time of listing and heart transplantation. | AA children faced higher waitlist mortality and rejection episodes post-heart transplantation compared to Caucasian counterparts, indicating disparities in transplant outcomes. |  |
| Holmes et al. [15] | Cross-Sectional Ecological Non-Experimental Study | USA | Patients only: AI/AN, Asian/Pacific Islander, Black AA, White | No specific tool – assumption from implicit assessment of racial bias by examining delivery methods’ impact on infant mortality across racial groups | Racial disparities in infant mortality rates associated with delivery method, with Black AA infants experiencing higher mortality rates regardless of delivery type. |  |
| Rambachan et al. [16] | Retrospective Study | USA | Patients only: White (45%), Asian (20%), Black (16%). LatinX (12%), Native Hawaiian or Other Pacific Islander (1%), AI or AN (1%), Other/Unknown (6%) | No specific tool – assumption from multivariable logistic regression assessing self-reported race/ethnicity’s association with 7-day readmission rates. | Black and Asian patients were more likely to be readmitted within 7 days post-discharge compared to White patients, despite adjusting for various demographic, clinical, and hospitalisation-based factors. |  |
| Nikpour et al. [17] | Descriptive, Correlation Design | USA | Patients only: NHW (75.9%), NHB/Hispanic/Non-Hispanic Other (24.1%) | No specific tool – assumption from examination of patient characteristics’ association with opioid and nonopioid prescriptions by race/ethnicity. | Disparities in opioid and nonopioid prescriptions among Veterans Affairs chronic pain patients based on race, gender, and education level, affecting pain management strategies. |  |
| Holt et al. [18] | Retrospective Observational Cohort Study | USA | Patients only: Black AA (20.6%), Non-Black AA (79.4%) – Female sex (57.8%) | No specific tool – assumption from comparison of hypertension treatment patterns in Black AA versus non-Black AA patients to assess impact of race-based guidelines. | Black AA patients prescribed thiazide diuretics and calcium channel blockers (CCB) more frequently as initial hypertension therapy yet experiencing higher rates of uncontrolled hypertension despite adherence to race-based guidelines. |  |
| Sinnott et al. [19] | Retrospective Observational Cohort Study | UK | Patients only: White, South Asian, Black, Other/Mixed/Unknown | No specific tool – assumption from analysis examining treatment outcome differences by race. | Both CCBs and angiotensin-converting enzyme inhibitors/angiotensin receptor blockers are effective in managing blood pressure. CCBs show greater efficacy in patients aged over 75, suggesting potential for simplifying guidelines. Healthcare providers should prioritise patient-specific health conditions over age and ethnicity when choosing hypertension medications. |  |
| **Perceived Discrimination and/or Medical Mistrust in Healthcare Access and/or Provision** | | | | | | |
| Cox et al. [20] | Cross-Sectional Sub-study | New York - USA | Patients only: Black (33.3%), White (26.8%), LatinX (24.6%), Another racial group(s) (15.3%, including Asian/Pacific Islander and Multiracial). | Group-Based Medical Mistrust Scale (GBMMS) and modified GBMMS-Sexual/Gender Minority (GBMMS-SGM). | Black sexual minority men reported higher levels of race-based and sexual/gender minority-based medical mistrust than White participants. |  |
| Devlin et al. [21] | Cross-Sectional Study | USA | Patients only: NHW (208), NHB (483), Hispanic (153). | Survey used included the Tuskegee Legacy Project's Likelihood of Participation scale and the Reactions to Race questions from the 2014 Behavioural Risk Factor Surveillance System (BRFSS) Questionnaire as well as analysis to evaluate associations among racial groups. | Perceived discrimination in healthcare negatively impacted willingness to participate in clinical research, with Black and Hispanic participants reporting higher discrimination but no significant differences in participation rates across racial/ethnic groups. |  |
| Pro and Zaller [22] | Cross-Sectional Study | USA | Patients only: White (78.86%), Black (9.21%), Latino/Latina (9.29%), AI/AN (2.64%) | No specific tool – assumption from multivariable logistic regression on weighted survey data from the National Epidemiologic Survey on Alcohol and Related Conditions-III to analyse past-year experiences of racial discrimination in healthcare settings, focusing on individuals with opioid use disorder and considering race/ethnicity and methadone maintenance treatment (MMT) status to identify potential racial bias. | MMT status was strongly linked to racial discrimination in healthcare, especially among AI/AN individuals, who had over thirty times the odds of discrimination if they had used MMT. |  |
| Walls et al. [23] | Community-Based Cross-Sectional Study | USA | Patients only: AI with Type 2 Diabetes Mellitus (T2DM) – 56% Female, 78% living on the reservation | Microaggressions assessed using questions adapted from the Racial Microaggression Counselling Scale. Depressive symptoms were measured using the PHQ-9 scale. | Over one-third of AI adults with T2DM experienced healthcare microaggressions, significantly correlating with poorer physical and mental health. |  |
| Hausmann et al. [24] | Cross-Sectional Mixed-Method Study | USA | Patients only: Non-Latino White (189 - 30.4%), non-Latino AA (235 - 37.8%), Latino (198 - 31.8%) | Everyday Discrimination Scale adaptation. | Negative interpersonal experiences and unresolved pain were major factors in perceived racial/ethnic discrimination among patients with pain, with significant dissatisfaction observed in Latinos (staff interactions), AA (staff demeanour), and Whites (unresolved pain). |  |
| **Providers Bias and Stereotyping** | | | | | | |
| Green et al. [25] | Retrospective Cross-Sectional Study | USA | Healthcare Professionals: 16,366 Physicians (8% Black, 71.5% White, 2.2% Hispanic, 17% Asian), 53,441 non-physician healthcare workers (NPHW) (13.9% Black, 75.7% White, 3.6% Hispanic, 4.6% Asian).  Patients: Black (12%), White (74.6%), Hispanic (5.8%), Asian (5.6%). | Race Implicit Association Test (IAT), Asian IAT, Native American IAT, Arab-Muslim IAT. | Both physicians and NPHW exhibited higher levels of prejudice against Black and Arab-Muslim individuals compared to the general population. Even after adjusting for demographics, NPHW still showed significant bias, with White NPHW displaying the most anti-Black prejudice. |  |
| Topaz et al. [26] | Retrospective Observational Cohort Study | USA | Healthcare Professionals: Nurses, Physical or Occupational therapist, Social Workers.  Patients: White (44%), Asian (9%), Hispanic (23%), Black (24%). | Natural Language Processing algorithm used to detect language of judgement in clinical notes. | Home care providers frequently use judgmental language, particularly with Hispanic and Black patients, who are 14% more likely to encounter this behaviour. This use of judgmental language is associated with a reduction in care visits by 21 minutes. |  |
| Won Yoo et al. [27] | Retrospective Cross-Sectional Study | Nevada - USA | Patients only: White (50.3%), Black (26.4%), Asian/Hawaiian/Pacific Islander (23.3%). Across all patient groups, 24.4% were Hispanic. | No specific tool – assumption from multivariate logistic regressions examined hypothesis that non-White patients were less likely to have 4Ms elements (What Matters, Mobility, Medication, and Mentation) documented compared to their White counterparts. | Disparities exist in the documentation of essential geriatric care components among different racial and ethnic groups, highlighting inequities in healthcare delivery. |  |
| Roach et al. [28] | Cross-Sectional Sub-study | Alberta - Canada | Healthcare Professionals only: Physicians | Implicit bias measured using the Indigenous-European IAT. Explicit bias measured via 2-feeling thermometer approaches. | Albertan physicians demonstrate explicit bias against Indigenous people, with 8.3% harbouring unfavourable feelings and a quarter preferring white individuals. Two-thirds of these physicians exhibit implicit bias, with white cisgender males showing the highest levels of bias. Notably, reverse racism was discussed in free-text responses, and discomfort was expressed with survey questions addressing bias and racism. |  |
| Hagiwara, Duffy and Quillin [29] | Cross-Sectional Study | North America -USA | Healthcare Professionals: 107 Cerified Genetic Counsellors.  Healthcare Students: 108 Genetic Counsellor Trainees. | Implicit racial bias measured using the Race IAT. Explicit racial bias measured using feeling thermometer. Implicit racial stereotyping measure using the Medical Cooperativeness IAT. Explicit racial stereotyping measuring using a 32-item measure designed to assess general racial stereotypes, and a 34-item measure to assess racial stereotypes in medical context. | Genetic counsellors, both certified and trainees, exhibit slight to moderate implicit racial bias favouring White Americans over Black Americans. However, they do not explicitly express prejudice or negative stereotypes. |  |
| Bunting et al. [30] | Randomised Vignette-Based Study | USA | Healthcare Students only: Medical School Students (788 Allopathic medicine, 684 Osteopathic Medicine). | IAT and Vignette-based test. | Medical students' assumptions about patients seeking pre-exposure prophylaxis (PrEP) for HIV are influenced by the race of the patient and their partner, illustrating how racial bias can impact healthcare perceptions. |  |
| Burton et al. [31] | Retrospective Qualitative Study | USA | Healthcare Professionals only: Physicians (AA (2.1%), Asian (26.2%), Hispanic or LatinX (3.7%), White (57.7%), Other (10.2%)). | No specific tool – assumption from analysis of race and gender trends in event reporting. | There is a lower threshold for reporting events involving physicians who are female and/or belong to a racial or ethnic minority group, indicating potential disparities in disciplinary actions based on identity. |  |
| Duveau et al. [32] | Prospective Quantitative Study with an Observational Design | Belgium | Healthcare Students Only: Postgraduate Medical School Students – General Practice Internship (second year Trainee General Practitioners (GPs). | IAT used with French-language and North African first names to measure implicit ethnic bias. Hudelson scale used to assess one dimension of cultural competence (responsibility for adapting care to migrant patients). | Trainee GPs in French-speaking Belgium showed moderate to strong implicit ethnic bias favouring their own ethnic group over North African ones. They are generally willing to adapt care for migrants but exhibit reluctance in situations involving language barriers or differing values. |  |
| van Andel et al. [33] | Double-Blind Randomised Controlled Trial | Netherlands | Healthcare Students: Medical School Bachelor and Mater Students.  Teachers included as an ecological valid choice. | Stereotypical Features Rating, Stereotypical Passage Text Rating, Inclusive Work Environment Scale, Social Dominance Orientation Scale completion following exposure to a picture stimulus. | Individual perceptions and preferences predict stereotypes, whereas social norms do not unintentionally reinforce stereotypes. |  |
| Puumala et al. [34] | Cross-Sectional Study | USA | Healthcare Professionals only: Physicians, Nurses, Advanced Practitioners – 62% Nurses – Over 90% identified as White and Non-Hispanic – 76.1% Female. | Implicit bias measure with a study specific IAT while explicit bias measured via statements on a five-point Likert scale for agreement. Clinical vignettes used to explore whether implicit and explicit bias among ED care providers influenced their clinical decision-making and treatment recommendations for paediatric patients based on race. | Most ED care providers exhibited implicit preference for NHW individuals over AI individuals. Explicit bias agreement ranges from 22% to 32%, affecting clinical decisions despite responses to clinical vignettes not showing significant bias effects. |  |
| van Ryn et al [35] | Prospective Observational Study | USA | Healthcare Students only: Medical School Students. | Black-White IAT. | Medical school experiences across formal curricula, informal curricula, and interracial contact domains were associated with changes in student implicit racial attitudes. Completing the Black-White IAT during medical school predicts decreased implicit racial bias. |  |
| D’Costa et al. [36] | Cross-Sectional Study | Australia | Healthcare Professionals only: All clinical and non-clinical staff of a cancer centre (including nurses, physicians, allied healthcare professionals, radiation therapist, surgeons, social workers, admin, human resources, engineers, executives, research, other clinical and other non-clinical staff). | Australian (Aboriginal-White) Race IAT. | Among employees at an Australian cancer centre, 60% displayed a preference for white Australians over First Nations Australians, with 21% showing moderate to strong preference. This indicates the presence of implicit racial bias potentially impacting patient care and outcomes. |  |
| Hymel et al. [37] | Retrospective Cross-Sectional Study | USA | Patients only: NHW (271), Minority Race/Ethnicity (229) | No specific tool – assumption from analysing frequencies and performed statistical comparisons between racial/ethnic groups, stratified by risk levels, and adjusted for relevant confounders to evaluate potential racial disparities in Abusive Head Trauma (AHT) reporting across the participating site. | Minority race/ethnicity patients were more frequently evaluated and reported for suspected AHT than white/non-Hispanic patients, particularly in lower risk cases, suggesting racial and ethnic disparities likely influenced by local physicians' implicit bias. |  |
| Blair et al. [38] | Prospective Cohort Study | USA | Healthcare Professionals: Primary care physicians (Female (54%), White (83%), >10years experience (49%)).  Patients: Black (982), Latino (1,484), White (2,328). | Implicit bias measure using the IAT. Additionally, analysis conducted to assess the relationship between clinicians' IAT scores and treatment intensification, medication adherence, and blood pressure control among Black and Latino hypertensive patients versus White patients. | Implicit racial/ethnic bias among primary care clinicians did not significantly affect treatment intensification, medication adherence, or blood pressure control among their Black and Latino patients with hypertension. |  |
| **Disparities in Healthcare Education and/or Training** | | | | | | |
| Pinder et al. [39] | Observational Study using Contemporaneous Data | UK | Healthcare Professionals only: UK medical graduates, 73 International medical graduates, 337 candidates from backgrounds other than medicine. | No specific tool – assumption from analysis of association between the psychometric test outcomes and demographic characteristics (including ethnicity) of the applicants. | Psychometric tests used in the selection process reveal unexplained variation, indicating differential attainment. Characteristics negatively associated with progression include being of Black or Asian ethnicity and coming from a non-UK medical graduate background. |  |
| Mukherji et al. [40] | Observational Cohort Study | UK | Healthcare Students only: Medical School Students (Non-White (2,134), White (1580)). | No specific tool – assumption from multinomial logistic regression performed to estimate the association between ethnicity and grade boundary attainment across all assessments, and linear regression used to estimate the association between ethnicity and examination marks, describing the change in attainment gap by calendar year and scholastic year. | Efforts to narrow the attainment gap in a UK medical school have not succeeded as White students consistently achieved higher grades compared to non-White students. This disparity persisted over time and notably widened over time for Black students. |  |
| Brown, Goss and Sam [41] | Retrospective Cohort Study | UK | Healthcare Students only: Medical School Students (BAME (30.9%), White (69.1%)). | No specific tool – assumption from analysis of UK Foundation Programme (UKFP) Z-scores at exit from medical school used to assess causes of Attainment Gap (AG) between BAME and White medical students, adjusting for several confounding factors. | Significant variations in UKFP Z-scores among ethnic subgroups were found, with BAME students on average having lower scores than White students, and the size of the AG varied considerably across medical schools. |  |
| **Disparities in Healthcare Literature** | | | | | | |
| Massie et al. [42] | Retrospective cross-sectional study | Global | Patients (Indirectly): analysed images of human skin in medical literature - White photo (78%), non-White photo (22%), White Graphics (95%), non-White graphics (5%). | Objective categorisation of skin tone in medical images, using the Fitzpatrick Scale as a proxy for race/ethnicity, and analysis of the proportional representation of these categorised images over time and by geographic region of the authors. | Medical literature, specifically in plastic surgery, does not accurately represent the racial diversity of patients. Despite an increase in the representation of non-White photographs over time and a stronger presence of diversity in articles by international authors. |  |
| Kalantari et al. [43] | Cross-sectional study | USA | Patients (Indirectly): analysed images of human skin in medical literature - White photo (77.6%), non-White photo (13.6%), Indeterminate (8.8%).  Healthcare Professionals (Indirectly): 29 images depicted clinicians - (75.7%) White, (13.8%) non-White, undetermined (10.3%). | Extraction of images with classification for type of image, race, and sex was completed independently by two reviewers, with a third reviewer available to resolve disagreements. The extraction tool was pilot tested before use. | There is a predominant representation of White individuals and males in the images across the two major emergency medicine textbooks analysed. This contrasts with the diverse demographics of patients seen in ED in the United States, highlighting a lack of representation and potential source of implicit bias in the hidden curriculum conveyed through these textbook visuals. |  |
| Rana et al. [44] | Cross-sectional study | USA | Patients (Indirectly): analysed images of human skin in medical literature (light skin tones (56.4%), medium (35.1%), dark (8.5%)). | New Immigrant Survey Skin Colour Scale used to assess images and grade skin tone. Each image was scored at least twice, with discrepancies resolved by a second board-certified dermatologist. | Published images of patients with lupus significantly overrepresent light skin tones and underrepresent dark skin tones, indicating a racial bias in medical educational materials that may limit care for patients with skin of colours who are at greater risk for complications from lupus. |  |
| Bell et al. [45] | Cross-sectional study | USA | Patients (Indirectly): analysed images of human skin in medical literature (dark skin (37), light skin (236)). | Objective categorisation of skin tone in medical images, using the Fitzpatrick Scale as a proxy for race/ethnicity, by two independent blinded reviewers, with a third available for disagreements. | A striking underrepresentation of dark skin tone images (only 13.6%) compared to light skin tones in the depiction of cutaneous disorders in top emergency medicine journals was found, with a high interobserver agreement on skin tone classification. |  |

**References:**

1. Holmes L, Enwere M, Mason R, et al. Medical Misadventures as Errors and Mistakes and Motor Vehicular Accidents in the Disproportionate Burden of Childhood Mortality among Blacks/African Americans in the United States: CDC Dataset, 1968–2015. *Healthcare*. 2024;12(4):477-477. doi:https://doi.org/10.3390/healthcare12040477

2. Rice CT, Barnett S, O’Connell SP, et al. Impact of gender, ethnicity and social deprivation on access to surgical or transcatheter aortic valve replacement in aortic stenosis: a retrospective database study in England. *Open Heart*. 2023;10(2):e002373. doi:https://doi.org/10.1136/openhrt-2023-002373

3. Sangal RB, Su H, Khidir H, et al. Sociodemographic Disparities in Queue Jumping for Emergency Department Care. *JAMA Network Open*. 2023;6(7):e2326338. doi:https://doi.org/10.1001/jamanetworkopen.2023.26338

4. Pacheco F, Luciano E, Hebert D, Serpa E, Solh W. Does race influence the attainment of the principles of oncologic surgical resection in colon adenocarcinoma? A Retrospective cohort analysis from the National Cancer Database. *Annals of Medicine and Surgery*. 2023;85(5):1562-1565. doi:https://doi.org/10.1097/ms9.0000000000000693

5. Johnson-Jennings M, Duran B, Hakes J, Paffrath A, Little MM. The influence of undertreated chronic pain in a national survey: Prescription medication misuse among American indians, Asian Pacific Islanders, Blacks, Hispanics and whites. *SSM - Population Health*. 2020;11:100563. doi:https://doi.org/10.1016/j.ssmph.2020.100563

6. Huang BB, Saseendrakumar BR, Delavar AG, Baxter SL. Racial Disparities in Barriers to Care for Patients With Diabetic Retinopathy in a Nationwide Cohort. *Translational Vision Science & Technology*. 2023;12(3):14-14. doi:https://doi.org/10.1167/tvst.12.3.14

7. Cascino T, Somanchi S, Colvin M, et al. Racial and Sex Inequities in the Use of and Outcomes After Left Ventricular Assist Device Implantation Among Medicare Beneficiaries. *JAMA network open*. 2022;5(7):e2223080. doi:https://doi.org/10.1001/jamanetworkopen.2022.23080

8. Longcoy J, Patwari R, Hasler S, et al. Racial and Ethnic Differences in Hospital Admissions of Emergency Department COVID-19 Patients. *Medical Care*. 2022;60(6):415-422. doi:https://doi.org/10.1097/mlr.0000000000001710

9. Liu TL, Taylor YJ, Schuch JC, Tucker L, Zager KM, Dulin MF. Variations in Receipt of Contraceptives by Insurance Status and Race/Ethnicity. *North Carolina Medical Journal*. 2022;83(1):58-66. doi:https://doi.org/10.18043/ncm.83.1.58

10. Javed Z, Maqsood MH, Amin Z, Nasir K. Race and Ethnicity and Cardiometabolic Risk Profile: Disparities Across Income and Health Insurance in a National Sample of US Adults. *Journal of Public Health Management and Practice*. 2022;28(1):S91-S100. doi:https://doi.org/10.1097/phh.0000000000001441

11. Basile Ibrahim B, Kennedy HP, Combellick J. Experiences of Quality Perinatal Care During the US COVID‐19 Pandemic. *Journal of Midwifery & Women’s Health*. 2021;66(5):579-588. doi:https://doi.org/10.1111/jmwh.13269

12. Agarwal S, Schechter C, Gonzalez J, Long JA. Racial-Ethnic Disparities in Diabetes Technology Use Among Young Adults with Type 1 Diabetes. *Diabetes Technology & Therapeutics*. 2021;23(4):306-313. doi:https://doi.org/10.1089/dia.2020.0338

13. Ali I, Vattigunta S, Jang JM, et al. Racial Disparities are Present in the Timing of Radiographic Assessment and Surgical Treatment of Hip Fractures. *Clinical Orthopaedics and Related Research*. 2020;478(3):455-461. doi:https://doi.org/10.1097/CORR.0000000000001091

14. Amdani S, Bhimani SA, Boyle G, et al. Racial and Ethnic Disparities Persist in the Current Era of Pediatric Heart Transplantation. *Journal of Cardiac Failure*. 2021;27(9):957-964. doi:https://doi.org/10.1016/j.cardfail.2021.05.027

15. Holmes L, O’Neill L, Elmi H, et al. Implication of Vaginal and Cesarean Section Delivery Method in Black–White Differentials in Infant Mortality in the United States: Linked Birth/Infant Death Records, 2007–2016. *International Journal of Environmental Research and Public Health*. 2020;17(9):3146. doi:https://doi.org/10.3390/ijerph17093146

16. Rambachan A, Abe-Jones Y, Fernandez A, Shahram Y. Racial Disparities in 7-Day Readmissions from an Adult Hospital Medicine Service. *Journal of Racial and Ethnic Health Disparities*. 2022;9(4):1500-1505. doi:https://doi.org/10.1007/s40615-021-01088-3

17. Nikpour J, Broome M, Silva S, Allen KD. Patient demographics and clinical characteristics influence opioid and nonopioid pain management prescriptions of primary care NPs, PAs, and physicians. *Journal of the American Association of Nurse Practitioners*. 2022;34(7):883-890. doi:https://doi.org/10.1097/jxx.0000000000000728

18. Holt HK, Gildengorin G, Karliner L, Fontil V, Pramanik R, Potter MB. Differences in Hypertension Medication Prescribing for Black Americans and Their Association with Hypertension Outcomes. *The Journal of the American Board of Family Medicine*. 2022;35(1):26-34. doi:https://doi.org/10.3122/jabfm.2022.01.210276

19. Sinnott SJ, Douglas IJ, Smeeth L, Williamson E, Tomlinson LA. First line drug treatment for hypertension and reductions in blood pressure according to age and ethnicity: cohort study in UK primary care. *BMJ*. 2020;371(8269). doi:https://doi.org/10.1136/bmj.m4080

20. Cox AB, Jaiswal J, LoSchiavo C, et al. Medical Mistrust Among a Racially and Ethnically Diverse Sample of Sexual Minority Men. *LGBT health*. 2023;10(6):471-479. doi:https://doi.org/10.1089/lgbt.2022.0252

21. Devlin A, Gonzalez E, Ramsey F, Esnaola N, Fisher S. The Effect of Discrimination on Likelihood of Participation in a Clinical Trial. *Journal of racial and ethnic health disparities*. 2020;7(6):1124-1129. doi:https://doi.org/10.1007/s40615-020-00735-5

22. Pro G, Zaller N. Interaction effects in the association between methadone maintenance therapy and experiences of racial discrimination in U.S. healthcare settings. *PLOS ONE*. 2020;15(2):e0228755. doi:https://doi.org/10.1371/journal.pone.0228755

23. Walls ML, Gonzalez J, Gladney T, Onello E. Unconscious Biases: Racial Microaggressions in American Indian Health Care. *The Journal of the American Board of Family Medicine*. 2015;28(2):231-239. doi:https://doi.org/10.3122/jabfm.2015.02.140194

24. Hausmann LRM, Jones AL, McInnes SE, Zickmund SL. Identifying healthcare experiences associated with perceptions of racial/ethnic discrimination among veterans with pain: A cross-sectional mixed methods survey. Laws MB, ed. *PLOS ONE*. 2020;15(9):e0237650. doi:https://doi.org/10.1371/journal.pone.0237650

25. Green TL, Vu H, Laura E.T. Swan, et al. Implicit and explicit racial prejudice among medical professionals: updated estimates from a population-based study. *The Lancet Regional Health - Americas*. 2023;21:100489-100489. doi:https://doi.org/10.1016/j.lana.2023.100489

26. Topaz M, Song J, Davoudi A, et al. Home Health Care Clinicians’ Use of Judgment Language for Black and Hispanic Patients: Natural Language Processing Study. *JMIR nursing*. 2023;6:e42552. doi:https://doi.org/10.2196/42552

27. Won Yoo J, Kang HT, Choe I, et al. Racial and Ethnic Disparity in 4Ms among Older Adults Among Telehealth Users as Primary Care. *Gerontology & geriatric medicine*. 2023;9:1-8. doi:https://doi.org/10.1177/23337214231189053

28. Roach P, Ruzycki SM, Hernandez S, et al. Prevalence and characteristics of anti-Indigenous bias among Albertan physicians: a cross-sectional survey and framework analysis. *BMJ Open*. 2023;13(2):e063178. doi:https://doi.org/10.1136/bmjopen-2022-063178

29. Hagiwara N, Duffy C, Quillin J. Implicit and explicit racial prejudice and stereotyping toward Black (vs. White) Americans: The prevalence and variation among genetic counselors in North America. *Journal of Genetic Counseling*. 2022;32(2):397-410. doi:https://doi.org/10.1002/jgc4.1648

30. Bunting SR, Feinstein BA, Calabrese SK, et al. Assumptions about patients seeking PrEP: Exploring the effects of patient and sexual partner race and gender identity and the moderating role of implicit racism. *PLOS ONE*. 2022;17(7):e0270861. doi:https://doi.org/10.1371/journal.pone.0270861

31. Burton É, Flores B, Jerome B, et al. Assessment of Bias in Patient Safety Reporting Systems Categorized by Physician Gender, Race and Ethnicity, and Faculty Rank. *JAMA Network Open*. 2022;5(5):e2213234. doi:https://doi.org/10.1001/jamanetworkopen.2022.13234

32. Duveau C, Demoulin S, Dauvrin M, Lepièce B, Lorant V. Implicit and explicit ethnic biases in multicultural primary care: the case of trainee general practitioners. *BMC Primary Care*. 2022;23(1). doi:https://doi.org/10.1186/s12875-022-01698-8

33. van Andel CEE, Born MP, van den Broek WW, Stegers‐Jager KM. Do norms unintentionally increase stereotypical expressions? A randomised controlled trial. *Medical Education*. 2021;56(3):331-338. doi:https://doi.org/10.1111/medu.14712

34. Puumala SE, Burgess KM, Kharbanda AB, et al. The Role of Bias by Emergency Department Providers in Care for American Indian Children. *Medical Care*. 2016;54(6):562-569. doi:https://doi.org/10.1097/mlr.0000000000000533

35. van Ryn M, Hardeman R, Phelan SM, et al. Medical School Experiences Associated with Change in Implicit Racial Bias Among 3547 Students: A Medical Student CHANGES Study Report. *Journal of General Internal Medicine*. 2015;30(12):1748-1756. doi:https://doi.org/10.1007/s11606-015-3447-7

36. D’Costa I, Hunt I, Russell L, Adams K. A racial bias test with tertiary cancer centre employees: why anti-racist measures are required for First Nations Australians cancer care equity. *Australian Health Review*. 2022;47(1):5-12. doi:https://doi.org/10.1071/AH21113

37. Hymel KP, Laskey AL, Crowell KR, et al. Racial and Ethnic Disparities and Bias in the Evaluation and Reporting of Abusive Head Trauma. *The Journal of Pediatrics*. 2018;198:137-143.e1. doi:https://doi.org/10.1016/j.jpeds.2018.01.048

38. Blair IV, Steiner JF, Hanratty R, et al. An Investigation of Associations Between Clinicians’ Ethnic or Racial Bias and Hypertension Treatment, Medication Adherence and Blood Pressure Control. *Journal of General Internal Medicine*. 2014;29(7):987-995. doi:https://doi.org/10.1007/s11606-014-2795-z

39. Pinder RJ, Bury F, Sathyamoorthy G, Majeed A, Rao M. Differential attainment in specialty training recruitment in the UK: an observational analysis of the impact of psychometric testing assessment in Public Health postgraduate selection. *BMJ Open*. 2023;13(3):e069738-e069738. doi:https://doi.org/10.1136/bmjopen-2022-069738

40. Mukherji P, Adas MA, Clarke B, et al. Changing trends in ethnicity and academic performance: observational cohort data from a UK medical school. *BMJ Open*. 2022;12(12):e066886. doi:https://doi.org/10.1136/bmjopen-2022-066886

41. Brown C, Goss C, Sam AH. Is the awarding gap at UK medical schools influenced by ethnicity and medical school attended? A retrospective cohort study. *BMJ Open*. 2023;13(12):e075945. doi:https://doi.org/10.1136/bmjopen-2023-075945

42. Massie JP, Cho DY, Kneib CJ, et al. Patient Representation in Medical Literature: Are We Appropriately Depicting Diversity? *Plastic and Reconstructive Surgery – Global Open*. 2019;7(12):e2563. doi:https://doi.org/10.1097/GOX.0000000000002563

43. Kalantari A, Alvarez A, Chung A, et al. Sex and Race Visual Representation in Emergency Medicine Textbooks and the Hidden Curriculum. *AEM Education and Training*. 2022;6(3). doi:https://doi.org/10.1002/aet2.10743

44. Rana A, Witt A, Jones H, Mwanthi M, Murray J, Zickuhr L. The Representation of Skin Colors in Images of Patients with Lupus Erythematosus. *Arthritis Care & Research*. 2021;74(11):1835-1841. doi:https://doi.org/10.1002/acr.24712

45. Bell G, Holmes S, Gillespie S, Wood A, Murray BL. Images of dark skin in top emergency medicine journals: A cross‐sectional analysis of images of emergent cutaneous disorders. *AEM Education and Training*. 2021;5(S1):S76-S81. doi:https://doi.org/10.1002/aet2.10683
